# Complementary roles for auxin and auxin signalling revealed by reverse engineering lateral root stable prebranch site formation

**DOI:** 10.1242/dev.200927

**Published:** 2022-11-21

**Authors:** Joana Santos Teixeira, Thea van den Berg, Kirsten ten Tusscher

**Affiliations:** Computational Developmental Biology Group, Faculty of Science, Utrecht University, Utrecht 3584 CH, The Netherlands

**Keywords:** Lateral roots, Prebranch sites, Priming, Auxin, Auxin signalling, Temporal integration

## Abstract

Priming is the process through which periodic elevations in auxin signalling prepattern future sites for lateral root formation, called prebranch sites. Thus far, the extent to which elevations in auxin concentration and/or auxin signalling are required for priming and prebranch site formation has remained a matter of debate. Recently, we discovered a reflux-and-growth mechanism for priming generating periodic elevations in auxin concentration that subsequently dissipate. Here, we reverse engineer a mechanism for prebranch site formation that translates these transient elevations into a persistent increase in auxin signalling, resolving the prior debate into a two-step process of auxin concentration-mediated initial signal and auxin signalling capacity-mediated memorization. A crucial aspect of the prebranch site formation mechanism is its activation in response to time-integrated rather than instantaneous auxin signalling. The proposed mechanism is demonstrated to be consistent with prebranch site auxin signalling dynamics, lateral inhibition, and symmetry-breaking mechanisms and perturbations in auxin homeostasis.

## Teaser

**Highlighted Article:** Computational modelling reveals the likely complementary roles of auxin and auxin signalling in one of the earliest steps in the formation of plant lateral roots, prebranch site formation.

## INTRODUCTION

The architecture of the plant root system – length of the main root, and number, length, positioning and angles of lateral roots (LRs) – determines its access to water and nutrients. As a consequence, root system architecture (RSA) is a major determinant of plant fitness and crop yields (Herder et al., 2010; Rogers and Benfey, 2015). Being the hidden half of the plant, root systems have been far less subjected to targeted breeding efforts. Optimization of crop root systems is therefore considered a major target for a next green revolution (Herder et al., 2010; Kong et al., 2014; Wollenweber et al., 2005). To achieve this, an in-depth understanding of the developmental processes shaping root system architecture and its adjustment to environmental conditions is needed. However, many aspects of RSA patterning remain to be revealed.

**Fig. 1.**
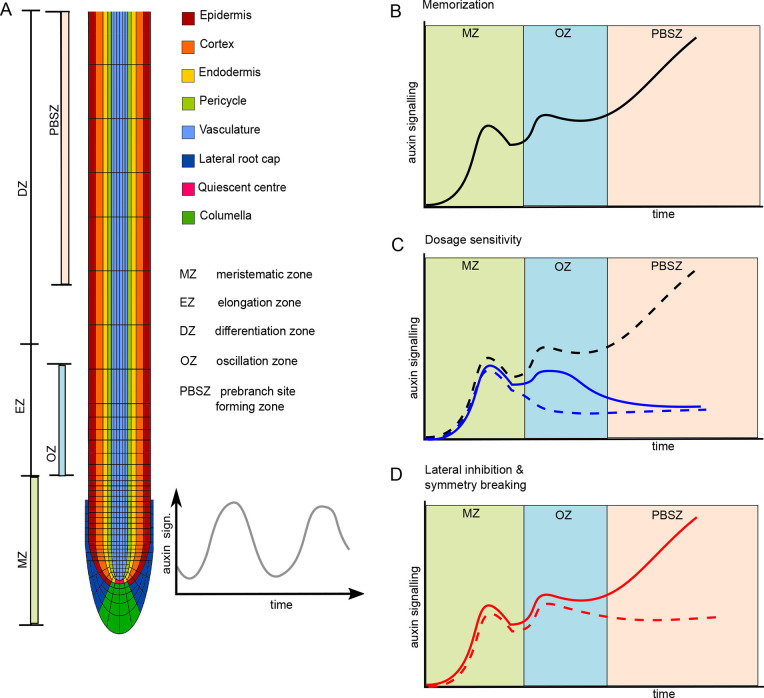
**Schematized depiction of experimentally observed auxin signalling characteristics during priming and PBS formation.** (A) Anatomy of the *Arabidopsis* root tip indicating the different cell types, with priming starting in the xylem pole vasculature and being transmitted to the pericycle, the developmental zones, with the MZ where cell divisions occur, the EZ where cells rapidly expand and the DZ where cells acquire their terminal cell fate, the oscillation zone (OZ) where priming occurs and the PBS-forming zone (PBSZ) where successful primings lead to stable PBS formation. (B) Cells undergoing successful priming experience high auxin signalling levels in the MZ, auxin signalling subsequently becomes high again in the OZ during priming, and after a second decline becomes elevated for the third time leading to stable PBS formation and thereby memorization of the transient priming signal. (C) Cells in between priming events (dashed blue line), as well as cells undergoing priming under limited auxin availability (solid blue line) experience lower OZ auxin signalling levels, and fail to establish a PBS. For comparison, a line representing cells undergoing successful priming is added (dashed black line). (D) Under normal conditions, lateral inhibition between nearby PBS-forming sites leads to establishment and maintenance of one and repression of the other PBS-forming site, preventing formation of multiple nearby PBSs. Solid line indicates auxin signalling levels at one xylem pole, dashed line auxin signalling levels at the opposite xylem pole.

A crucial aspect of RSA patterning is the formation of LRs. LR development starts with a process called priming, the prepatterning of sites competent for future LR formation. This process entails periodic oscillations in auxin and/or auxin signalling at the start of the elongation zone (EZ) (Fig. 1A) (De Smet et al., 2007; Moreno-Risueno et al., 2010; Xuan et al., 2015, 2016), which through growth become translated into a spatially periodic pattern of LR-competent sites (Moreno-Risueno et al., 2010; Xuan et al., 2015, 2016). Only if priming has sufficient amplitude is it successful, leading to the formation of a stable prebranch site (PBS) (Fig. 1B,C) (Xuan et al., 2015), after which founder cell identity establishment, LR initiation and subsequent LR development, emergence and outgrowth may occur (Malamy and Benfey, 1997).

Although for these later stages of LR formation the involved regulatory modules, generally centred around auxin signalling, and downstream processes have been well documented (Du and Scheres, 2018; Lavenus et al., 2013; Santos Teixeira and Ten Tusscher, 2019), we have very limited knowledge about the earliest stages. The mechanism underlying LR priming has long remained enigmatic, with hypotheses ranging from gravitropic bending (De Smet et al., 2007) and genetic oscillators (Moreno-Risueno et al., 2010), to periodic shedding of the LR cap (Xuan et al., 2016). Recently, we demonstrated that priming may arise through a reflux-and-growth mechanism (van den Berg et al., 2021). We revealed a crucial role for tip-driven root growth, resulting in periodic variations in the sizes and therefore passive auxin uptake capacity of cells arriving in the elongation domain. Combined with root tip auxin reflux, resulting in an auxin-loading domain in the EZ, this gives rise to periodic oscillations in auxin levels in elongating cells.

However, it remains unclear how these transient priming events in the EZ are transduced into the next stages of LR formation occurring in the differentiation zone (DZ). This implies that the transient priming signal is somehow stably memorized and at a later stage sets in motion LR initiation. The observation that in successful priming auxin signalling levels first decline before rising again (Fig. 1B) (Xuan et al., 2015) further supports the notion that this memorization involves processes distinct from the initial priming (Laskowski and Ten Tusscher, 2017). Additionally, although priming occurs at both xylem poles in *Arabidopsis* and initially involves a larger area, wild-type LR formation is typically constrained to a single side and location (Fig. 1D). The existence of mutants in which nearby LR formation does occur (Chang et al., 2015; Fernandez et al., 2020; Hofhuis et al., 2013; Maule et al., 2013; Toyokura et al., 2019) implies the existence of active lateral-inhibition and symmetry-breaking mechanisms. Some of this inhibition occurs prior or during stable PBS formation (el-Showk et al., 2015; Toyokura et al., 2019). Finally, stable PBS formation is highly dosage sensitive, with low amplitude oscillations failing to produce PBSs, but an absence of auxin negative-feedback mechanisms resulting in supernumerous PBSs that subsequently fail to develop into LRs (Perianez-Rodriguez et al., 2021; Xuan et al., 2015). In summary, the mechanism responsible for transducing bilateral priming events into unique, single-sided stable PBSs in a dosage-dependent manner has so-far remained elusive.

A major factor hampering progress in our understanding of early LR formation is its limited observability. Markers exist for later stages, with, for example, founder cells being demarcated by GATA23 (GATA TRANSCRIPTION FACTOR 23) and MAKR4 (MEMBRANE-ASSOCIATED KINASE REGULATOR 4) expression, and ACR4 (ARABIDOPSIS CRINKLY 4) and RALFL34 (RALF-LIKE 34) involved in the formative divisions of LR initiation (De Rybel et al., 2010; De Smet et al., 2008; Murphy et al., 2016). Developing LRs can be distinguished based on typical root patterning transcription factors, such as WOX5 (WUSCHEL-RELATED HOMEOBOX 5), SHR (SHORT ROOT), SCR (SCARECROW) and the PLTs (PLETHORAs) (Du and Scheres, 2017) and can also be microscopically discerned based on morphological characteristics (Malamy and Benfey, 1997). In contrast, neither reporters nor anatomical markers exist to distinguish primed from non-primed pericycle cells. Although single-cell transcriptomics may eventually allow subpopulations of xylem pole pericycle cells to be distinguished, given the low numbers of primed cells and the complex spatiotemporal patterning this is far from trivial. As a consequence, it remains hard to determine experimentally how successfully primed cells differ from non-primed cells, and how these differences contribute to subsequent LR development.

In this study, we extend our model for LR priming to investigate how priming leads to stable PBS formation, a first prerequisite for LR initiation. We reverse engineer a hypothetical PBS formation mechanism that relies on temporal integration of the transiently occurring priming signal with auxin-induced upregulation of auxin signalling, and auxiliary roles for auxin-induced upregulation of auxin transport and production. To determine the plausibility of the proposed mechanism, we assess its compatibility with key characteristics of priming and PBS formation, lateral inhibition, symmetry breaking, and spatial narrowing of the auxin signalling domain. Our hypothesized PBS formation mechanism suggests that from a very early stage onwards successfully primed cells should become epigenetically distinct from their surroundings. Additionally, by revealing an essential role for priming-driven upregulation of auxin signalling capacity, the proposed PBS mechanism reconciles previously diverging viewpoints on the importance of auxin levels versus auxin signalling in early LR prepatterning.

## RESULTS

### Priming signal minimally maintained in pericycle cells

**Fig. 2.**
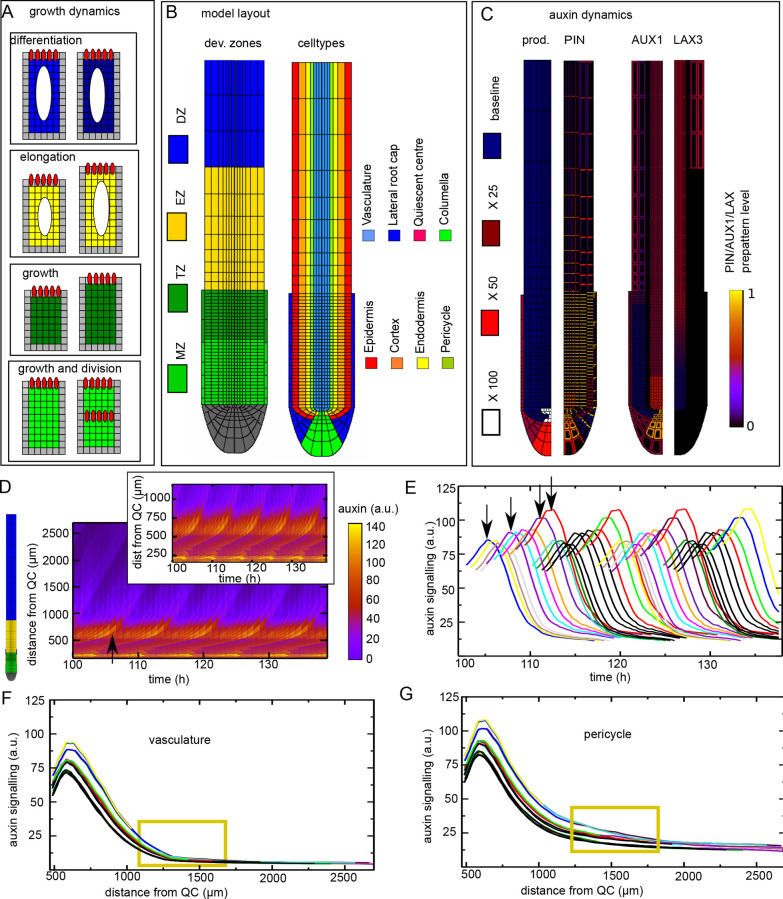
**Baseline model setup and priming dynamics.** (A) Model growth dynamics, with cell divisions in the MZ (light green), slow cytoplasmic growth but no divisions in the transit-amplifying zone (TZ; dark green), rapid cell expansion in the EZ (yellow) and terminal differentiation of cells in the DZ (blue). (B) Model root tip layout with on the left the location of the distinct root developmental zones and on the right the cell types making up the root tip, similar to Fig. 1A. (C) Model auxin dynamics arise from cell type- and developmental zonation-specific prepatterns for auxin production rates, PIN auxin exporter, and AUX1 and LAX3 auxin importers. For model details, see Materials and Methods. (D) Kymograph showing the spatiotemporal dynamics of auxin signalling in the pericycle for five sequential priming events. Snapshot on the left shows developmental zonation to enable interpretation of the distance from QC. Inset shows the same kymograph zoomed in on the lowermost parts of the root tip for comparison with our previous results (Van den Berg et al., 2021). (E) Auxin signalling dynamics as a function of time for a series of pericycle cells traced over time once they reach a distance of 500 from the root tip. Traced cells correspond to cells involved in the second to fifth priming event in D, with each priming event consisting of ten cells. Arrows indicate the first, fifth, ninth and tenth cell of the first priming sequence. (F) Auxin signalling as a function of distance for the vascular cells neighbouring the pericycle cells shown in B. (G) Auxin signalling dynamics as a function of distance for the same pericycle cells as shown in E. In E-G, line colour is based on the order in which cells reach a distance of 500 from the root tip. To limit the number of colours used, colours were re-used for cells at sufficient temporal distance ensuring that individual cellular auxin signalling dynamics could be discerned.

Using a computational modelling approach (Fig. 2A-C), we recently we uncovered a mechanism for LR priming, the prepatterning of pericycle cells competent for future LR formation (van den Berg et al., 2021). Briefly, this model contained realistic root tip growth dynamics, with slow stem cell divisions and rapid transit amplifying divisions in the meristem, and cell elongation and differentiation in more shootward zones (Fig. 2A), as well as realistic auxin dynamics, with elevated production of auxin around the stem cells, in the columella and LR cap, and cell and developmental zone-specific patterns of the AUX/LAX auxin importers and PIN auxin exporters (Fig. 2B,C). We demonstrated how these two ingredients suffice to generate oscillations in auxin dynamics, with root tip polar auxin transport producing an auxin-loading zone at the start of the EZ, and root growth generating periodic variations in the size at which cells enter this zone, causing periodic variations in auxin loading potential particularly in narrow vasculature cells with high surface to volume ratios (van den Berg et al., 2021). Combined, these mechanisms generate the oscillations in vascular auxin levels that underlie LR priming. Here, we built on this earlier model to investigate how auxin dynamics during priming may lead to stable PBS formation. To achieve this, we incrementally adjusted our earlier model. As a first model adjustment, we endow our new root tip model with an extended DZ and highly regular division dynamics (see Materials and Methods). This enables us to follow auxin dynamics over a prolonged spatiotemporal interval (Fig. 2D, kymograph), and ensures regular, reproducible priming dynamics (Fig. 2E) (Movie 1). This regularity allows us to only display dynamics in the first, fifth, ninth and tenth cell of the first priming event shown in Fig. 2E (arrows) in later figures. To validate the robustness of our results, we confirmed that for staggered rather than parallel cell wall positioning similar priming dynamics occur (Fig. S1, see Materials and Methods). Importantly, in our baseline model, for simplicity auxin signalling levels are equated to auxin levels (see Materials and Methods).

When looking at auxin flux direction (Fig. S2A), we observed that as EZ cells elongate epidermal and cortical fluxes reorient from predominantly upward to upward and inward, whereas endodermal fluxes change from downward to inward, together generating an auxin flux towards pericycle and vasculature cells. For cells arriving largest, this transition occurred closest to the start of the EZ (Fig. S2A, compare cells marked by yellow stars with those marked by orange and red stars), consistent with the enhanced auxin loading potential of these cells uncovered previously. However, although cell sizes keep increasing and, once switched, flux directions remain constant until cells enter the DZ, auxin levels in primed cells quickly dissipated (Fig. 2D,E; Movie 1). We hypothesized that this dissipation is due to the general decline in auxin levels that occurs as cells become displaced to positions further away from the root tip as a result of the growth and division of younger cells below them. To investigate this, we stopped root growth after a priming event, resulting in auxin levels being maintained rather than dissipating (Fig. S2B).

Fig. 2F,G shows that although similar auxin dynamics occur in the vasculature and pericycle, in the pericycle the cells that receive the highest auxin levels were able to maintain their elevated auxin levels to a limited extent, but such maintenance was absent in the vasculature (yellow boxes). This can be understood from the more apoloar endodermal and cortical PIN patterns in the DZ (see Materials and Methods). Indeed, from the auxin flux directions we observed that in the DZ the vasculature flux orients towards the pericycle, resulting in a passing on of remaining auxin to the pericycle. Additionally, endodermis and pericycle fluxes became oriented towards one another, giving rise to a recycling of auxin previously shown to contribute to maintaining and enhancing pericycle auxin levels (Laskowski et al., 2008). However, final EZ and DZ pericycle auxin signalling levels were far below those observed *in planta* during PBS formation, which typically are similar to auxin signalling levels during priming.

### Direct positive feedback fails to differentially maintain auxin signalling in primed cells

**Fig. 3.**
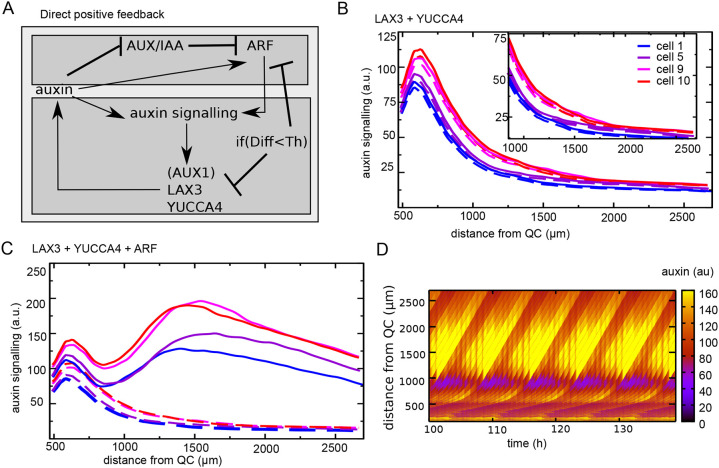
**Positive feedback generates a secondary auxin signalling response.** (A) Overview of added regulatory interactions to add positive feedback and enhanced auxin signalling capacity. Auxin signalling upregulates the expression of LAX3 and YUCCA4, as well as ARF, with this upregulation being repressed below a certain cellular differentiation level (indicated by Diff<Th). AUX1 and LAX3 expression occurs in all cells, whereas YUCCA4 and ARF are restricted to the EZ and DZ to ensure activation in response to priming but not due to high meristematic auxin levels. Additionally, YUCCA4 expression is limited to the vasculature and pericycle based on experimental data (Tang et al., 2017). (B) Pericycle auxin signalling when only the LAX3+YUCCA4 positive-feedback loop is added. Auxin dynamics is shown for the first, fifth, ninth and tenth cell in a priming event; dashed lines indicate auxin signalling dynamics in the absence of these feedback loops (maximum expression level of LAX3 and YUCCA4=100, Km=20). (C) Pericycle auxin signalling when both feedback loops are added (maximum expression level LAX3 and YUCCA4=100, maximum expression ARF=300, Km=30); auxin signalling in the absence of these feedback loops is shown with dashed lines. (D) Kymograph of pericycle auxin signalling corresponding to the settings in C.

*In planta*, auxin signalling levels in primed cells are observed to first decline and then rise again, suggesting a second, separate mechanism for stable PBS formation (Laskowski and Ten Tusscher, 2017). Positive feedback would be a logical mechanism to amplify and maintain differences in auxin levels and/or signalling between cells. Indeed, auxin-dependent induction of auxin importers, such as AUX1 (AUXIN RESISTANT 1) and LAX3 (LIKE AUX1 3) (Laskowski et al., 2008), as well as a LEC2 (LEAFY COTELYDON 2)- and FUS3 (FUSCA3)-induced upregulation of YUCCA4 mediating auxin biosynthesis (Tang et al., 2017), have been implicated in early stage LR formation. The initial decline and subsequent rise of auxin signalling implies that these positive-feedback mechanisms do not contribute to the priming-induced initial auxin elevation, but instead need to become activated with a certain delay. Elevated cytokinin signalling in the early EZ (Dello Ioio et al., 2007) could be a potential candidate for the initial suppression of these positive-feedback mechanisms. As a next step, we therefore extended our model with additional auxin signalling-dependent LAX3 as well as YUCCA4 expression (we ignored the intermediary factors LEC2 and FUSCA3). To achieve a secondary rise of auxin signalling in the absence of explicitly modelled cytokinin signalling dynamics, we simply assumed that this LAX3 and YUCCA4 expression can only occur once cells have reached a particular developmental stage (Fig. 3A), which occurs at approximately 880 μm from the root tip, when the original priming signal is halfway through its decline.

Even when applying a low auxin induction threshold value and/or high maximum expression levels for LAX3 and YUCCA4, only a very limited secondary enhancement of auxin signalling levels was observed (Fig. 3B, Fig. S3A,B). Previously, it has been suggested that auxin signalling rather than auxin levels increase substantially as part of priming (Moreno-Risueno et al., 2010). We therefore hypothesized that enhancement of auxin signalling capacity could help to generate an increase in auxin signalling despite an overall decrease in auxin levels, and that ARF (AUXIN RESPONSE FACTOR) 5, 7, 19, which are known to play crucial roles during early LR formation (Lee et al., 2009; Du and Scheres, 2018; Lavenus et al., 2013), may require transcriptional upregulation. Next, we therefore included an auxin-inducible generic ARF in our model, again gated to become expressed only beyond a specific cellular developmental stage (Fig. 3A). In essence, this resulted in a priming-induced increase in the efficiency of how auxin levels are translated into auxin signalling strength. The resulting auxin signalling level in this model is composed of the sum of baseline auxin signalling, equated to auxin levels (note that we do not explicitly model ubiquitously expressed ARFs), and additional priming-induced auxin signalling, for which we do explicitly model ARF, AUX/IAA and auxin interactions (see Materials and Methods). We observed that the enhanced auxin signalling capacity does indeed induce a significant secondary increase in auxin signalling levels (Fig. 3C,D; Movie 2). However, because auxin signalling ultimately depends on auxin availability, auxin signalling eventually still declined. Additionally, primed and non-primed cells showed qualitatively similar behaviour in terms of this secondary increase in auxin signalling, which is inconsistent with experimental results. This qualitative behaviour was independent of the precise maximum level of ARF expression and its auxin induction threshold (Fig. S3C,D). Finally, although additional LAX3 and induction of YUCCA4 expression quantitatively contributed to a secondary rise in auxin signalling, ARF induction alone was sufficient (Fig. S3E).

### Temporal integration of priming signal robustly discerns primed cells

A recent study on phyllotaxis, the auxin-driven periodic patterning of leaves in the plant shoot apex, demonstrates that it is a temporally integrated rather than instantaneous auxin signal that drives the auxin-dependent transcription of developmental genes (Galvan-Ampudia et al., 2020). The authors suggest that one likely mechanism for such temporal integration of auxin signalling is through epigenetic state changes. Inspired by this, we decided to incorporate into our model the chromatin state of a not yet specified gene, calling it EpiO, where low values indicate a closed and high values an open chromatin state. Experimental data indicate that auxin-dependent TIR1/AFB signalling, through the degradation of Aux/IAA and resulting liberation of ARF, results in the recruitment of chromatin modifiers that enhance an open chromatin state (Lee et al., 2017; Wu et al., 2015), with opposing factors contributing to a closed chromatin state (Fukaki et al., 2006). Based on this, we assume a basal closed chromatin state, with chromatin opening increasing with auxin signalling level. Additionally, chromatin closing is assumed to decrease with both auxin signalling level and chromatin open state, as the latter counteracts chromatin closing through the resulting gene expression (see Materials and Methods).

**Fig. 4.**
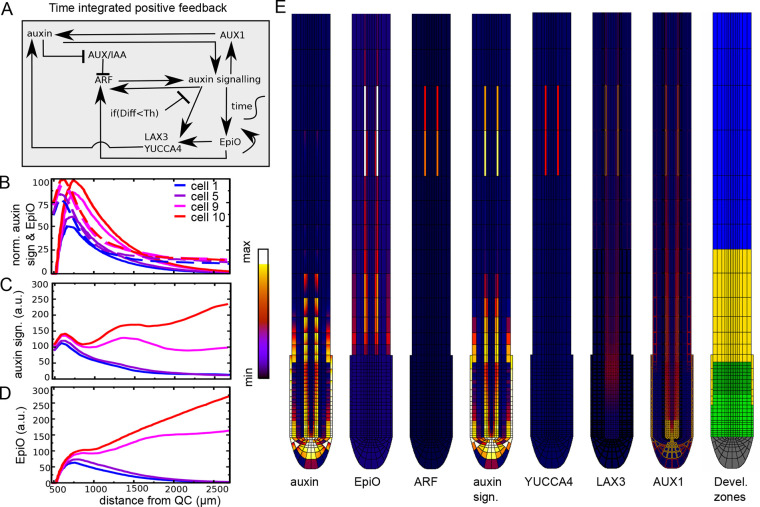
**Time-integrated positive feedback enables stable PBS formation.** (A) Overview of regulatory interactions in the model settings where positive feedback on ARF, LAX3 and YUCCA3 is gated by the EpiO state. The EpiO state arises from temporal integration of the auxin signalling level (indicated by the integral sign with time as label). Again, auxin signalling-driven upregulation of ARF, LAX3 and YUCCA3 expression is repressed below a certain cellular differentiation level (Diff<Th). (B) Comparison of (normalized) auxin signalling (dashed lines) and EpiO (solid lines) dynamics for default parameter settings and in the absence of positive feedback. (C,D) Pericycle auxin signalling (C) and EpiO (D) dynamics as a function of distance in the combined EpiO+positive feedback model. (E) Snapshot of auxin concentration, EpiO state, ARF, auxin signalling, YUCCA4 and LAX3 levels and developmental zonation patterns illustrating the induction of a high EpiO state resulting in high ARF, YUCCA and LAX3 expression and high auxin signalling levels in two pairs of cells that have undergone priming.

First, we studied EpiO dynamics in the absence of additional LAX3, YUCCA4 or ARF expression. Comparing normalized auxin signalling and EpiO dynamics, we observed that for sufficiently slow dynamics the EpiO state is capable of accumulating the smaller, instantaneous auxin signalling differences occurring at each time instance into a difference in chromatin open state that is significantly larger (see Fig. S4A,B for non-normalized auxin and EpiO dynamics). For default parameter settings, peak normalized auxin signalling differed by 25 a.u. (100 versus 75), whereas peak EpiO levels differed by 54 a.u. (100 versus 46), a more than twofold increase in differences (Fig. 4B). Additionally, we observed that EpiO differences are significantly longer maintained. At a distance of 1000 μm from the root tip, auxin signalling differences declined to 17 a.u. (45 versus 28), whereas EpiO levels still differed by 44 a.u. (70 versus 26), a 2.5-fold increase in differences (Fig. 4B). This longer maintenance is promoted by high EpiO levels slowing down EpiO decay, representing transcriptional activity slowing down chromatin closing. Thus, particularly at the later stages relevant for PBS formation, EpiO levels are more suited to differentiate primed cells and selectively amplify and memorize auxin signalling in these cells.

Precise build-up and maintenance of the chromatin open state depends on parameter values. More rapid EpiO dynamics resulted in more rapid EpiO build-up and hence stronger amplification of auxin signalling differences, at the cost of maintenance (Fig. S4C). In contrast, lowering the auxin signalling threshold for chromatin opening (EpiO build-up) enhanced both maximum amplitude and maintenance of EpiO (Fig. S4D). In the absence of data, parameter values were chosen such that build-up and prolonged maintenance of EpiO signal is well supported.

### Temporal integration-based positive feedback maintains auxin signalling in primed cells

Having established the suitability of an auxin-dependent chromatin open state in discerning primed from non-primed cells, we then reinstated the previous positive-feedback mechanisms. We assume that the EpiO state gates the auxin-dependent expression of ARF, LAX3 and YUCCA4, allowing for their transcription only beyond a certain level of chromatin opening (see Materials and Methods). In the case of LAX3, we assume EpiO only gates the priming-related part of its expression, but not the broader, more general expression of LAX3 in the vasculature (el-Showk et al., 2015). Again, to ensure a secondary, delayed increase in auxin signalling after priming, ARF, LAX3 and additional YUCCA4 expression only occurs once cells have reached a particular developmental stage (Fig. 4A).

Fig. 4C-E shows that by using EpiO to gate LAX3, YUCCA4 and ARF expression, a much more selective enhancement of auxin signalling only in cells receiving high auxin levels during priming occurs (Movie 3). Indeed, although ARF expression can be induced in any tissue type, it was selectively induced only in primed pericycle cells, enabling the highly specific upregulation of auxin signalling and downstream responses (Fig. 4E). Additionally, because of the additional positive feedback now present between EpiO status and ARF expression, a stably maintained, continuous rising of auxin signalling level occurred, consistent with experimental observations (Fig. 4C). Finally, and interestingly, for the applied parameter settings auxin signalling in the cell experiencing the second highest auxin levels during priming also underwent this continuous rise, albeit at lower levels. Stable PBS formation could be restricted to a single cell (albeit still on both sides of the root) by decreasing the maximum level (Fig. S5A), or increasing the activation threshold (Fig. S5C) of ARF expression, whereas increasing the auxin signalling activation threshold for LAX3 and YUCCA4 had a negligible effect on auxin signalling dynamics (Fig. S5E,F). Finally, although additional LAX3 and induction of YUCCA4 contribute significantly, ARF induction alone was again sufficient to drive a secondary rise in signalling (compare Fig. S4C with Fig. S5G).

**Fig. 5.**
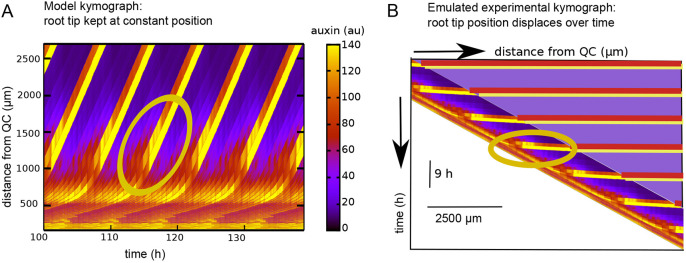
**Narrowing and increase of auxin signalling during PBS formation.** (A) Standard model kymograph for the model simulation shown in Fig. 4C-E. (B) Kymograph emulating experimental kymographs. The kymograph was obtained by skewing the kymograph in A such that individual cells in the DZ are maintained at a constant horizontal position reflecting the absence of growth in this region. This automatically results in the continuous downward displacement of the lower parts of the root tip in which growth occurs. To further enhance the resemblance, we artificially added what the kymograph would look like if in our model no culling of cells at the top of the simulation domain would occur but instead an increasingly large domain would be simulated as the root grows. Yellow ovals highlight the narrowing (reduction from high auxin in two cells to one cell) and increasing (increase in auxin level in remaining high auxin cell) auxin signalling.

An important hallmark of priming and stable PBS formation is that although initially auxin signalling occurs in a relatively broad domain during and directly after priming, the auxin signalling domain subsequently narrows and signalling intensity increases, settling into a narrow auxin signalling peak demarcating a stable PBS (Moreno-Risueno et al., 2010). To investigate whether our proposed PBS formation mechanism is consistent with these dynamics, we examined in detail the spatiotemporal auxin signalling dynamics (Fig. 5). To illustrate more clearly how the kymographs in our model are related to the auxin signalling space-time plots commonly applied in experimental papers on LR priming (Duan et al., 2021; Kircher and Schopfer, 2018; Xuan et al., 2015, 2016), we show both our standard model kymograph, in which root tip position is fixed at the base of the diagram (Fig. 5A), and a transformation thereof, in which root position moves downward through growth and in which the constant, finite size simulation domain is artificially complemented to generate a kymograph as though the simulation domain were allowed to grow continuously (Fig. 5B). In both graphs (inside yellow ovals), we observed that initially a group of approximately five cells experienced an intermediate level of auxin elevation (red) that over time narrowed down to two cells experiencing higher auxin levels (yellow), finally narrowing to a single cell as one of these cells decreased its auxin levels (orange), consistent with the experimentally observed spatial narrowing of auxin signalling (Moreno-Risueno et al., 2010; Xuan et al., 2016).

### Lateral inhibition and symmetry breaking determine final PBS spacing

**Fig. 6.**
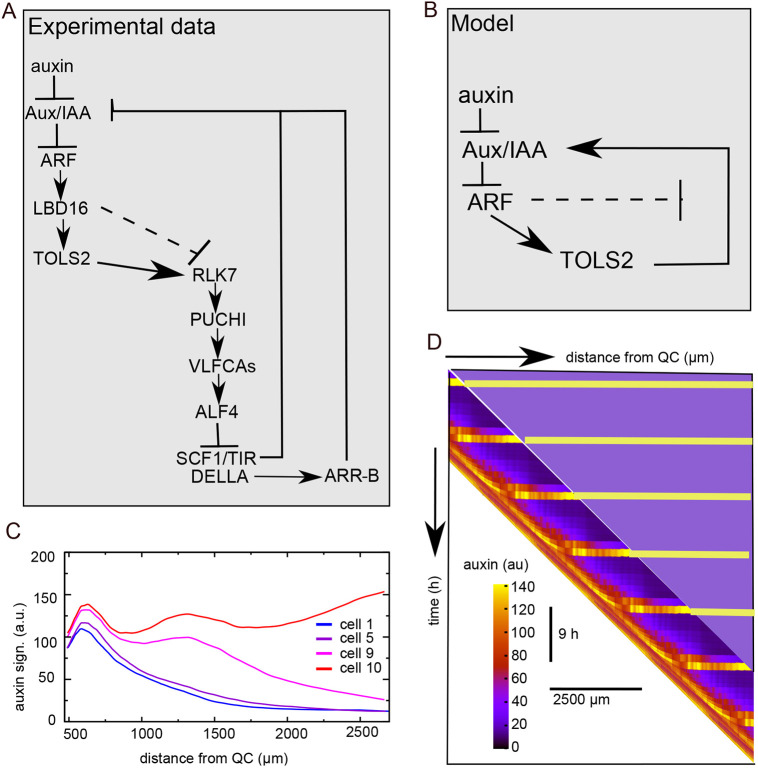
**TOLS2 signalling-mediated lateral inhibition.** (A) Layout of the network of experimentally uncovered regulatory interactions between LBD16 (LATERAL BOUNDARY DOMAINS 16), TOLS2 (TARGET OF LBD SIXTEEN 2), RLK7 (RECEPTOR LIKE PROTEIN KINASE 7), PUCHI, VLFCAs, ALF4 (ABERRANT LATERAL ROOT FORMATION PROTEIN 4), SCF/TIR, DELLA and AUX/IAA. For lateral inhibition to work, TOLS2 signalling should repress auxin signalling in neighbouring cells, but not in the cell itself, suggesting that a thus-far-undiscovered interaction exists that represses RLK7 signalling in high auxin signalling cells (dashed interaction). (B) Simplified TOLS2 network incorporated in the root tip model. (C) Pericycle auxin signalling dynamics in the model with TOLS2 signalling. (D) Experimental style type kymograph of pericycle auxin signalling dynamics of the same model simulation.

*In planta*, the formation of multiple nearby PBSs is prevented through a range of lateral inhibition-type mechanisms. Described ACR4, PLETHORA, and plasmodesmatal effects on repression of nearby LR formation are only activated at or after the LR initiation stage (De Smet et al., 2008; Hofhuis et al., 2013; Maule et al., 2013), whereas symmetry breaking due to auxin-dependent expression of AUX1 (el-Showk et al., 2015), the LBD16-TOLS2-RLK7-PUCHI pathway (Fig. 6A) (Toyokura et al., 2019) and possibly also cytokinin signalling-mediated repression (Chang et al., 2015) are active from earlier stages onward. To investigate the compatibility of our proposed PBS formation mechanism with early-stage lateral inhibition, we kept the parameter settings from Fig. 4, whereby a secondary PBS with lower auxin signalling was formed, and investigated how this secondary PBS can become repressed.

Auxin-dependent AUX1 (and LAX3) expression is already part of our baseline model. Therefore, we added a simplified version of the LBD16-TOLS2-RLK7-PUCHI pathway to our model. For this pathway to function as a lateral inhibition mechanism, PUCHI should become selectively activated in cells neighbouring the high-auxin, LBD16-expressing cells, but not in these cells themselves through a thus-far-unidentified mechanism we approximate here as an auxin-mediated inhibition of PUCHI (Fig. 6A,B). Additionally, PUCHI activation should result in a reduction of auxin levels or signalling capacity. Experimental data have shown that PUCHI results in enhanced production of very long chain fatty acids (VLFCAs) (Trinh et al., 2019), which have furthermore been shown to upregulate ALF4 (Shang et al., 2016), a repressor of SCF/TIR1 and SCF/SLY (Bagchi et al., 2018), ultimately resulting in the upregulation of AUX/IAA, both directly through SCF/TIR1 downregulation, as well as indirectly via SCF/SLY1 downregulation of DELLA that, via ARR-B, leads to further upregulation of AUX/IAA. Assuming that these downstream effects of PUCHI take place in cells neighbouring the high-auxin and high-LBD16-expressing cells, this results in a downregulation of auxin signalling in these cells. Fig. 6 shows that, for sufficiently strong TOLS2 signalling, inclusion of this mechanism enables the rootward stronger PBS to repress the shootward weaker PBS (Fig. S6, Movie 4), consistent with experimental observations (Toyokura et al., 2019). Note that because of the mutual repression, overall auxin signalling levels became somewhat lower also in the remaining stable PBS.

**Fig. 7.**
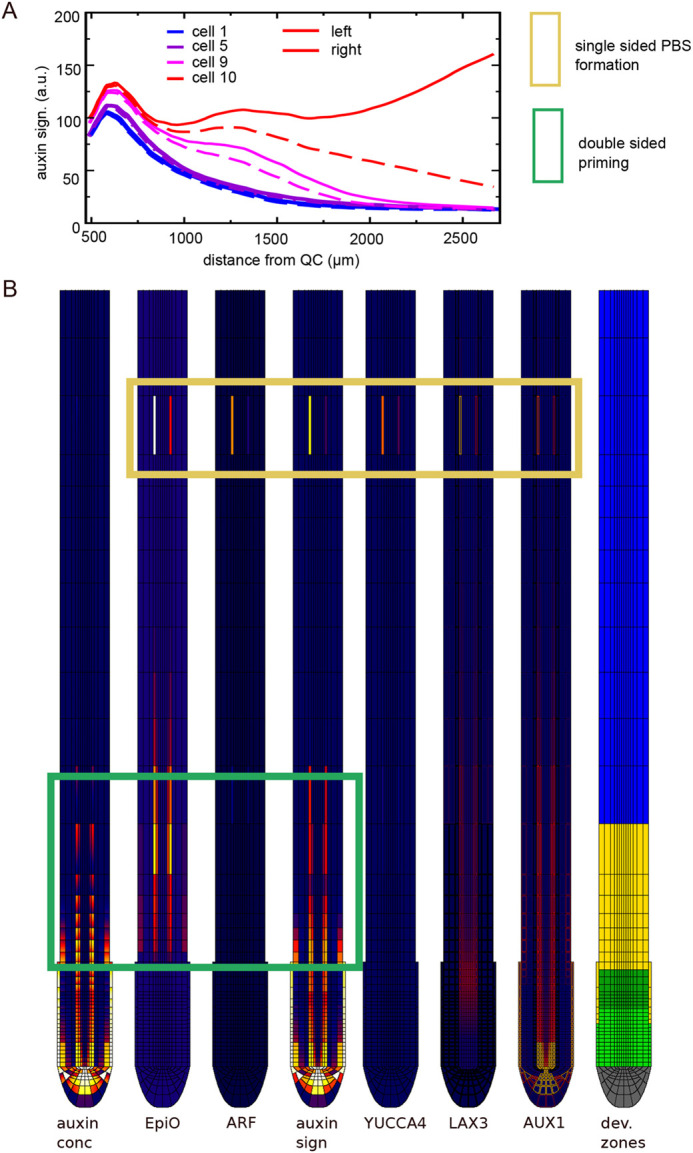
**PBS symmetry breaking.** (A) Auxin signalling dynamics in left (solid lines) and right (dashed lines) xylem pole pericycle cells. (B) Snapshot of auxin, EpiO, free ARF, auxin signalling, YUCCA4 expression, LAX3 and AUX1 membrane levels and developmental zonation. The lower part of the snapshots shows symmetric priming (green box) and the top part shows asymmetric PBS formation (yellow box). See key in Fig. 4C.

Earlier modelling studies have shown that the competition for auxin resulting from lateral-inhibition mechanisms results in amplification of initial small differences in auxin level, either resulting from fluctuations in auxin concentration (el-Showk et al., 2015) or curvature-induced cell size differences (Laskowski et al., 2008), leading to symmetry breaking between left and right PBSs. Similar effects can be expected in the stochastic differences in the sizes of left and right xylem pole pericycle cells, their expression of auxin importers and exporters, as well as root bending. Here, we incorporated a limited level (10%) of initial asymmetry in AUX1 expression to explore whether symmetry breaking would occur. We found that although a significant enhancement of the initial asymmetry occurs, with the shootward, weaker PBS being repressed more rapidly and the remaining, rootward PBS obtaining lower auxin signalling levels at the low AUX1 side (Fig. S7A), no full repression of PBS at the weaker AUX1 side occurred. If we additionally changed the affinity of AUX1 from 75 to 85 Km, making it less auxin sensitive, we did observe a complete repression of PBS at the weaker AUX1 side (Fig. 7A), where an initial small asymmetry during double-sided priming was translated into single-sided PBS formation (Fig. 7B, Movie 5). Full symmetry breaking could also be obtained by elevating the affinity of LAX3 or enhancing the effect of TOLS2 signalling (Fig. S7B,C), supporting the idea that these inhibitory mechanisms act additively. Additionally, similar results were obtained for imposing an initial asymmetry in, for example, LAX3 instead of AUX1 (Fig. S7D). Finally, our model incorporates a weak, radial distance-dependent and concentration difference-dependent auxin flow between corresponding positions in the left and right side of the root (see Materials and Methods) to take into account the 3D nature of actual plant roots. To investigate the importance of this 3D auxin flow coupling, we either removed or enhanced this coupling (Fig. S8A,B), demonstrating that this reduces or enhances, respectively, the symmetry breaking between opposite-sided PBSs, supporting the relevance of 3D auxin flows.

### Dosage dependence and importance of auxin homeostasis for priming

**Fig. 8.**
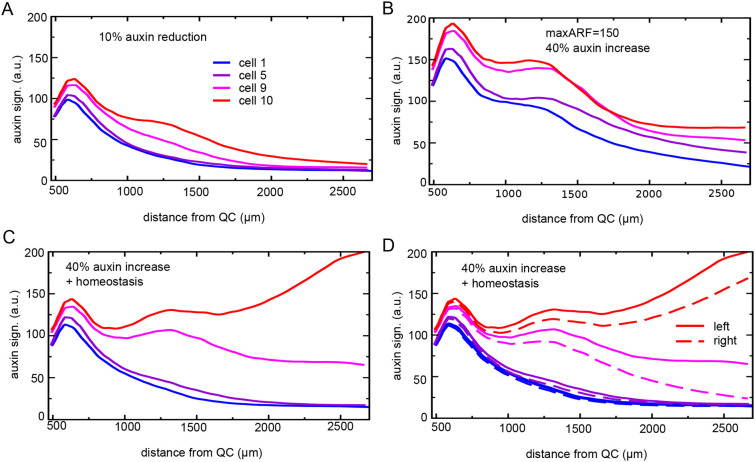
**Auxin dosage affects priming effectiveness.** (A) A reduction of 10% in root tip auxin content reduces priming amplitude and abolishes stable PBS formation. (B) A 50% reduction of maximum ARF levels combined with a 40% increase in root tip auxin generates transient secondary elevations in auxin signalling in most cells. (C,D) Combining a 40% increase in auxin inflow with an auxin homeostasis mechanism restores the selective secondary auxin signalling elevation in two consecutive cells and the subsequent lateral inhibition between them (C), yet does not fully restore left-right symmetry breaking (D).

*In planta*, owing to stochasticity in root bending, cell growth, division, gene expression, etc., there is inherent variability in auxin levels between different priming events. In case overall auxin levels are high, most priming events result in sufficiently high auxin signalling levels to lead to stable PBS and LR formation. In contrast, if auxin availability is lowered through, for example, mutants in which the production of the auxin precursor IBA in the LR cap is reduced, average priming amplitude is decreased, and only the higher amplitude subset of priming events results in stable PBS formation (Xuan et al., 2015). In our deterministic model, this variability between priming events was absent and we instead simply demonstrated that a lowering of auxin levels results in failure of PBS formation (Fig. 8A, compare with Fig. 7A).

In addition to low auxin availability being detrimental, it was recently shown that elevated auxin levels may also significantly reduce LR numbers. Mutations in either ARF7 or IAA18/POTENT (auxin insensitive mutant) were shown to result in elevated auxin levels, an increase in PBS formation and subsequent failure of LR formation (Perianez-Rodriguez et al., 2021). These mutations were interpreted as defects in the root clock, an alternative mechanism proposed for priming. In contrast, we argue that in the absence of an experimentally reported mechanism for cell-autonomous gene expression oscillations and considering the clear effects on auxin levels, these mutants should be considered as defects in root auxin homeostasis. To investigate this hypothesis, we simulated a single *potent* or *arf7* mutant in our model through a decreased expression of the generic ARF (50%), assuming that although ARF7 is non-functional, other ARFs, such as ARF19, are still functioning, as well as an increased auxin availability as a result of this mutation (influx increased by 40%). Fig. 8B shows that most cells, rather than only those experiencing the highest initial auxin signalling levels, generate a secondary response, consistent with the experimental observation of prolific PBS formation in *potent*/*arf7* mutants. Interestingly, this secondary response was more modest and in later stages dissipated, with even cells experiencing the highest initial auxin signalling not able to mount the continuous increase in auxin signalling observed for wild-type conditions. Again, this is consistent with observations for the *potent/arf7* mutants to fail in subsequent LR development (Perianez-Rodriguez et al., 2021). Our results indicate that  a lower auxin signalling capacity cannot be simply compensated for by more auxin. Given that auxin itself spreads out due to transport, whereas auxin signalling is cell bound, instead more cells undergo a less effective secondary auxin signalling response. To support further this possible role of excess auxin underlying the *potent/arf7* phenotype, we next added both the extra auxin as well as a simplified, ARF-dependent auxin degradation to our simulation with normal, wild-type ARF levels (see Materials and Methods). Under these conditions, the secondary increase in auxin signalling indeed remains restricted to the two highest auxin experiencing cells at each root side, with subsequent lateral inhibition between these two sites (Fig. 8C). However, not only is lateral inhibition slightly slowed at the high-auxin side compared with earlier (Fig. 8C), left-right symmetry breaking is also not fully restored (Fig. 8D). Our results thus support the suggestion that auxin homeostasis is essential to prevent precocious yet non-effective PBS formation, but also indicate that *in planta* auxin homeostasis is spatiotemporally more refined than currently incorporated in our model.

### Generality of PBS formation mechanism

**Fig. 9.**
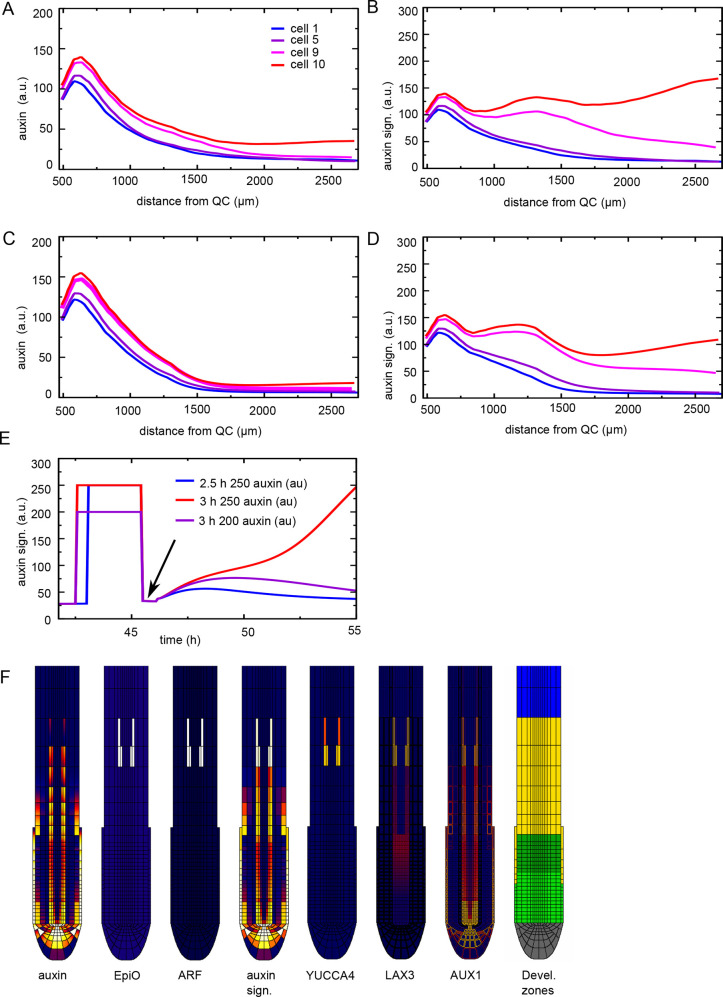
**Robustness of PBS mechanism.** (A,B) Pericycle auxin concentration (A) and auxin signalling (B, identical to Fig. 4C) dynamics as a function of distance in the EpiO model in absence of AUX1 asymmetry and TOLS2-mediated lateral inhibition. (C,D) Pericycle auxin concentration (C) and auxin signalling (D) dynamics in the same model as in A except for endodermis and cortex PIN patterns now being the same in the EZ and DZ. (E) Auxin signalling dynamics in a model without growth for imposed auxin signals of various strengths and durations. Arrow indicates the end of superimposed auxin signal and start of model endogenous auxin dynamics. (F) Snapshot of auxin, EpiO, free ARF, auxin signalling, YUCCA4 expression, LAX3 and AUX1 membrane levels and developmental zonation in the non-growing model when subjected to a 3 h pulse of 250 a.u. auxin at the 55 h time point shown in E. See key in Fig. 4C.

Finally, we set out to establish the robustness of model outcomes to some further changes in model assumptions. In Fig. 2F, we demonstrated a limited maintenance of auxin signalling in primed pericycle cells caused by the presence of a more apolar DZ PIN pattern. To address whether this is necessary for PBS formation, we ran a simulation in which endodermal and cortical PIN patterns were kept identical between EZ and DZ (Fig. 9). For simplicity, this was done in the absence of AUX1 asymmetry and TOLS2 signalling, as these processes modulate auxin levels and thus potentially obscure the effects of different DZ PIN patterns. We found that for a non-apolar DZ PIN pattern auxin levels in the shootward part of the root were lower (compare Fig. 9C and 9A), resulting in reduced auxin signalling strength during PBS formation (compare Fig. 9D and 9B). However, qualitatively similar dynamics were observed as in the default model.

Finally, we have thus far modelled our PBS formation mechanism as being triggered by our previously described reflux-and-growth priming mechanism (van den Berg et al., 2021). To establish the generality of the proposed PBS mechanism, we decided to instead superimpose an elevation in auxin levels and monitor whether stable PBS formation still occurs. To block reflux-and-growth priming, which arises as an emergent property from auxin reflux and root growth dynamics, we performed simulations in absence of root growth. Additionally, for simplicity, symmetry breaking and lateral inhibition were again ignored. The absence of growth-mediated cell displacement abolished the normally occurring decrease in cellular auxin levels over time, causing the intermediate level auxin concentrations of the early EZ over the long term to result in EpiO activation, and thereby spurious PBS formation. To prevent this, we blocked EpiO activation at the start of the EZ. Without any further changes in model parameters, we subsequently imposed an auxin pulse in the shootward part of the EZ. We found that for a sufficiently high level (250 a.u.) and long-lasting (3 h) auxin pulse, a persistently increasing secondary auxin signalling response resembling our earlier results arose (Fig. 9E,F), supporting the generality of the proposed PBS formation mechanism.

## DISCUSSION

In the current work, we extended our previous modelling efforts to elucidate the earliest steps of LR. Previously, we elucidated a so-called reflux-and-growth mechanism for LR priming (van den Berg et al., 2021). We demonstrated how the root tip auxin reflux loop establishes an auxin loading domain at the start of the EZ, which, combined with growth-driven periodic variations in cell sizes and hence auxin loading capacity, result in semi-periodic oscillations in cellular auxin levels. However, reflux-and-growth priming results in only transient increases in auxin levels, leaving open the question of how successful priming is transduced into stable PBS formation as characterized by a secondary stable peak of auxin signalling (Moreno-Risueno et al., 2010).

Here, using a computational reverse-engineering approach, we established the essential ingredients for stable PBS formation. First, we established that incorporating positive feedback through auxin-dependent expression of auxin-importing and -biosynthesizing proteins is insufficient for generating a significant secondary rise in auxin signalling. We demonstrated that an auxin-dependent upregulation of ARF genes enables increases in auxin signalling capacity, thereby counteracting the growth-driven dissipation of auxin levels in primed cells. Additionally, we found that responding to a temporally integrated auxin level, assumed to arise from auxin-dependent chromatin opening, enables a robust distinction between primed and non-primed cells. Combined, these two processes enable specifically those cells experiencing the highest auxin levels to mount a secondary response that increases over time, in agreement with experimental observations (Toyokura et al., 2019; Xuan et al., 2015). The mechanism proposed here bears close resemblance to observations made on phyllotaxis, in which a temporally integrated auxin signal was shown to induce the transcriptional response of cells (Galvan-Ampudia et al., 2020), and to recent findings on fractal floral architectures where a key role was identified for stable memorization of transient LEAFY expression (Azpeitia et al., 2021).

The proposed PBS reproduces the experimentally observed spatial narrowing and simultaneous increase in auxin signalling as a stable PBS is formed and is consistent with previously identified mechanisms for lateral inhibition and symmetry breaking that translate double-celled, double-sided priming into a single-celled, single-sided PBS. In the current model, we incorporated only auxin-dependent AUX1 and LAX3 and the ARF-LBD16-TOLS2-RLK7-PUCHI inhibitory mechanisms. However, given, for example, the convergent nature of the TOLS2-RLK7-PUCHI and GLV6/GLV10-RGI peptide signalling pathways (Jourquin et al., 2022), one could interpret the mechanism implemented here rather as a proxy for a combination of *in planta* mechanisms. Although the mechanisms considered in our model act during the translation from priming signal to stable PBSs, other inhibitory mechanisms, such as those mediated by ACR4, PLETHORA and plasmodesmatal dynamics (De Smet et al., 2008; Hofhuis et al., 2013; Maule et al., 2013), play a role at later stages. This is consistent with our findings that, depending on the strength of AUX1/LAX3/PUCHI mechanisms, early-stage lateral inhibition may be incomplete, thus requiring later-stage inhibitory mechanisms.

We believe that the mechanism proposed here offers a reconciliation of the dispute regarding whether oscillatory LR prepatterning involves elevations in auxin concentration or auxin signalling (Moreno-Risueno et al., 2010). Our model suggests that although priming involves an elevation of auxin concentration, to become translated into a stable PBS a subsequent elevation in auxin signalling is essential. This corresponds to the previously suggested distinction between priming signal and memory formation (Laskowski and Ten Tusscher, 2017). Furthermore, assuming that ARF7 is (one of) the ARF(s) upregulated by priming, our model explains the observed oscillations in ARF7 expression (Moreno-Risueno et al., 2010). The failure of out-of-phase addition of auxin to result in ARF7 expression and additional PBS formation was previously argued to imply that auxin alone is insufficient for PBS formation (Moreno-Risueno et al., 2010). Our results suggest that the auxin application was possibly too limited to change epigenetic state and enable PBS formation. Additionally, the involvement of lateral-inhibitory mechanisms may well explain the failure of additional PBS formation. Finally, it was recently shown that in both ARF7 and IAA18 (*potent*) mutants, auxin levels and the number of PBSs increased, but subsequent LR development was blocked (Perianez-Rodriguez et al., 2021). Interpreting these mutants as auxin homeostasis rather than clock mutants, and simulating this through addition of auxin, we demonstrated that excess auxin enabled ARF7 activation and PBS formation in most cells, but individually these cells reached lower auxin signalling levels, explaining supernumerous PBS formation and subsequent halting of further LR development.

In conclusion, our results suggest that although the priming signal consists of an elevation in auxin concentration, its conversion to a PBS involves a time-integrated measuring of this auxin and a subsequent translation into increased auxin signalling. Assuming this time integration has an epigenetic basis, epigenetic marks on auxin signalling factors involved in PBS formation could serve as an early marker distinguishing these from other pericycle cells. Our work demonstrates that auxin levels and signalling capacity cannot be traded against each other, thus defining separate essential roles for auxin and auxin signalling. Sufficient auxin during priming is needed to activate an enhanced auxin signalling capacity, with this triggered auxin signalling subsequently enabling further LR development.

## MATERIALS AND METHODS

### Root anatomy, cell types and developmental zones

We simulate root growth, auxin and gene expression dynamics on a realistic root tip topology, similar to previous studies (van den Berg et al., 2016, 2021; Cruz-Ramírez et al., 2012; Di Mambro et al., 2017; Salvi et al., 2020). However, in contrast to these earlier studies, in order to follow what happens over time to primed sets of pericycle cells and track whether these develop into stable PBSs, we model a significantly larger part of the DZ (Fig. S1A). This enables us to follow these cells as over time as, owing to growth and division of new cells, their distance from the root tip increases and they occupy a position further and further into the DZ.

Our model includes from inside to outside epidermis, cortex, endodermis, pericycle and vascular cell types. Additionally, in the root tip it incorporates LR cap, columella and quiescent centre (QC) cell types (Fig. S1A). In addition to cell types, developmental zones are included. From the root tip shootwards, we include the meristem proper, in which cell divisions occur; the transition zone, in which cells no longer divide but still undergo slow cytoplasmic growth; the EZ, in which cells undergo rapid vacuolar expansion; and the DZ, in which cells attain their final differentiation characteristics (Fig. S1A). For simplicity, the location and size of these zones are superimposed in this model.

### Auxin dynamics

Our root topology is laid out on a grid. Individual grid points belonging to the root tissue either correspond to the inside of a cell, a cell membrane or a cell wall. Except for the cells in the lowermost, curved part of the root tip, cells are modelled as rectangles of grid points. Cells have a cell type-specific width, whereas cell height is a function of growth stage and developmental zone, similar to our previous models (van den Berg et al., 2021; Salvi et al., 2020). Because of this grid-level subcellular resolution, our model enables the simulation of intracellular and intra-apoplastic auxin diffusion and the resulting gradients.

Intracellular auxin dynamics are described using the following partial differential equation:
(1)

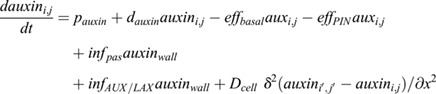
where *p*_*auxin*_ is auxin production, *d*_*auxin*_ auxin degradation, *eff*_*basal*_ basal ABCB-mediated auxin efflux, *eff*_*PIN*_ PIN-mediated auxin efflux, *inf*_*pas*_ passive auxin influx, *inf*_*AUX*/*LAX*_ AUX/LAX-mediated influx and *D*_*cell*_ intracellular auxin diffusion.

For auxin production, *p*_*auxin*_, in addition to a baseline production rate, *p*_*auxin*,*baseline*_, we incorporate the experimentally observed higher production of auxin around the QC and in the columella, as well as the high production of auxin precursors in the LR cap (Fig. S1B). For this, we multiply the baseline production rate with a parameter *celltypefactor*, which equals 100 for the QC, top columella layer and vascular initials, 50 for the lower columella layers and 30 for the LR cap; for all other cell types, *celltypefactor* is set to 1 (see Table 1). Additionally, based on its reported relevance for very early stages of LR development (Tang et al., 2017), we incorporate YUCCA4 expression-dependent auxin production, which, through the parameter *yucca*4*factor*, is translated into an increase of auxin production rate. Parametrization is carried out such that at a maximum YUCCA4 expression level of 100, auxin production rate increases 16.25-fold (Table 1). Combined, this gives rise to the following expression for auxin production:
(2)

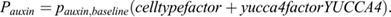
Because auxin production is applied per grid point, we finally normalize this auxin production relative to cell height to ensure that overall auxin production of a cell is not a function of cell height, by multiplying the above term with 
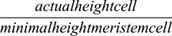
.

**Table 1.**
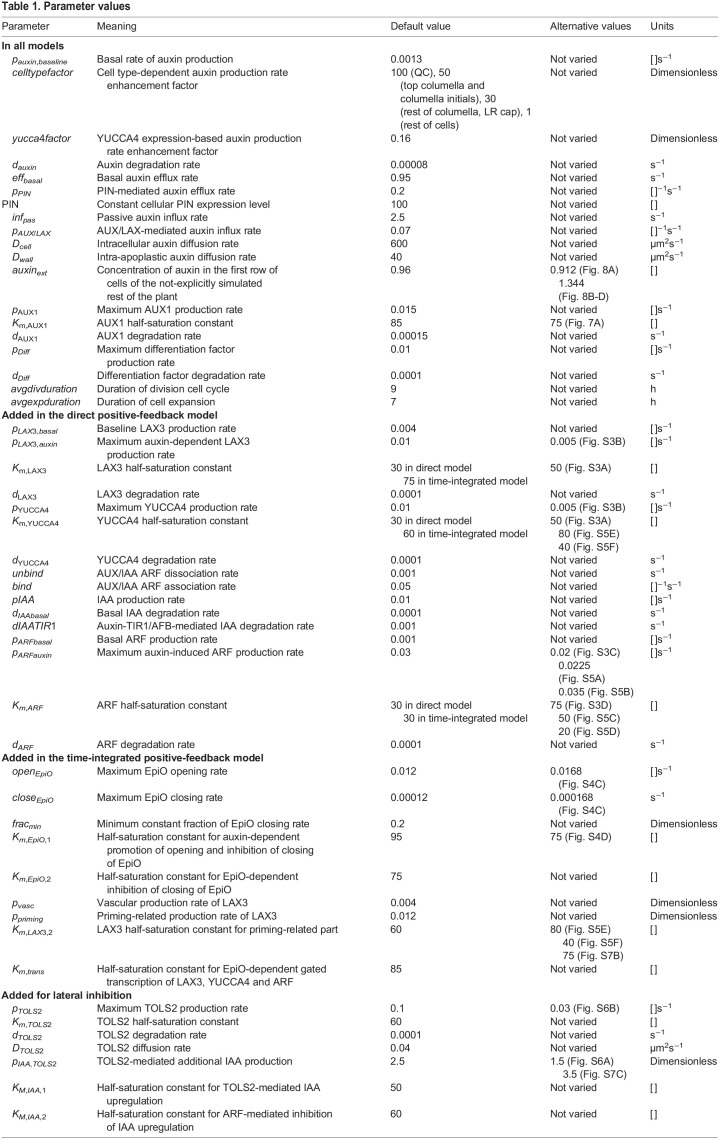
Parameter values

For PIN-mediated auxin efflux, *eff*_*PIN*_, we incorporate a cell- and zone-type PIN prepattern defining the maximum expression levels and polarity pattern (Fig. S1C). Actual membrane PIN levels are defined as the product of these membrane grid-level prepattern levels and cellular PIN expression level, which in this model for simplicity we assumed to be constant (at a level of 100):
(3)

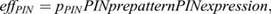
where *p*_*PIN*_ is the rate of PIN-mediated transport.

Similarly, for AUX/LAX-mediated auxin influx, *inf*_*AUX*/*LAX*_, we incorporate cell- and zone-type-specific prepatterns for AUX1 and LAX3 (Fig. S1D,E), whereas actual membrane AUX1 and LAX3 levels are defined as the product of prepattern level and gene expression level. AUX1 expression is standard auxin dependent; for model extensions auxin signalling dependent LAX3 expression is added (see below). Overall active influx is defined as the sum of AUX1 and LAX3 mediated influx:
(4)

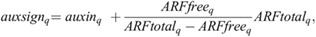
where *p*_*AUX*/*LAX*_ is the rate of AUX/LAX-mediated transport.

Expression of AUX1 is auxin dependent in all model settings (see below); in the baseline model, LAX3 expression is set constant at a level of 40, and in the direct and time-integrated feedback model settings it is auxin dependent (see below).

Values for the parameters discussed above and in the next sections can be found in Table 1.

### Auxin signalling dynamics

In our baseline model, to reduce model complexity, we do not explicitly model AUX/IAA and ARF dynamics and instead equate auxin signalling levels to cellular auxin levels, similar to various other studies (van den Berg et al., 2016; Laskowski et al., 2008; Mironova et al., 2012). It is noteworthy that although the translation from cellular auxin levels, via SCF/TIR1-mediated AUX/IAA degradation, non-linearly translates into free ARF levels, the subsequent translation to gene expression levels is also non-linear and their combined effects can be easily captured by a non-linear dependence of gene expression directly on cellular auxin levels. We assume that this baseline auxin signalling-induced gene expression relies on ARFs generally expressed throughout the root.

In the model settings incorporating the priming-dependent induction of ARF expression, we model overall cellular auxin signalling level as the sum of auxin levels (i.e. baseline auxin signalling, as before) and additional auxin signalling:
(5)

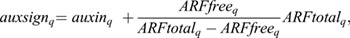
where *ARFtotal*_*q*_−*ARFfree*_*q*_ equals the amount of bound ARF and the second term represents the additional signalling taking place as the product of the total added auxin signalling capacity (*ARFtotal*, i.e. the amount of expressed ARF) multiplied by a fraction of free versus bound ARF reflecting the actual use of this additional capacity.

### Boundary conditions

Similar to previous root tip models developed by us as well as others (van den Berg et al., 2021; Grieneisen et al., 2007; Mähönen et al., 2014), we simulate auxin exchange with the not-explicitly modelled rest of the plant. For this, we model an inflow of auxin into the walls above the topmost vascular, pericycle and endodermal cells, and an outflow of auxin from the walls above the topmost epidermal and cortical cells. The inflow term consists of a constant, low, external auxin concentration (*auxin*_*ext*_) multiplied by the efflux (basal and PIN mediated) of not-explicitly simulated cells one layer above the topmost simulated cells, and which is taken to be equal to the efflux of these topmost simulated cells. The outflow term consists of the local wall auxin concentration multiplied by 10% of the influx (passive and AUX/LAX) of not-explicitly simulated cells, which is taken to be equal to the influx of the topmost simulated cells.

### Gene expression dynamics

Gene expression dynamics are modelled at the cellular level, using ordinary differential equations. For simplicity, we do not distinguish separate transcription and translation dynamics, but only describe resulting protein dynamics.

#### Baseline model

In our baseline model, following our earlier work (van den Berg et al., 2021), we incorporate the auxin-dependent expression of the auxin importer AUX1:
(6)

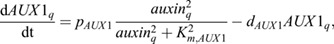
where *p*_AUX1_ is production rate, *K*_m,AUX1_ the half-saturation constant of auxin dependent transcription, and *d*_AUX1_ degradation rate.

To describe the differentiation dynamics cells undergo upon entering first the elongation and subsequently the DZ, we describe the dynamics of a generalized transcription factor as follows:
(7)

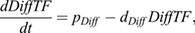
where *p*_*Diff*_ is the production rate of this differentiation factor, which is set to zero as long as cells are in the meristematic zone (MZ) and obtains a non-zero value upon EZ entry, and *d*_*Diff*_ is the degradation rate of this differentiation factor, which in the absence of ongoing differentiation (i.e. if cells were to revert to the MZ owing to changes in hormone or gene expression levels) allows for dedifferentiation.

#### Direct positive-feedback model

As a next step, we extended the model with additional positive-feedback regulations known or suggested to be involved in the earliest stages of LR formation.

First, the auxin importer LAX3 is also known to have auxin-dependent expression (Fig. 3A). We thus incorporated:
(8)

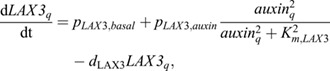
where *p*_*LAX*3,*basal*_ is the basal production rate, *p*_*LAX*3,*auxin*_ the maximum auxin-dependent additional production rate, *K*_m,LAX3_ the half-saturation constant of auxin-dependent transcription, and *d*_LAX3_ the degradation rate.

Second, in early LR formation, pericycle-specific expression of the transcription factors LEC2 and FUS3 has been shown to induce the auxin-biosynthesizing enzyme YUCCA4 (Tang et al., 2017). Additionally, expression of FUS3, and indirectly also LEC2, is auxin dependent (Gazzarrini et al., 2004; Horstman et al., 2017). Combined, this gives rise to another positive-feedback loop (Fig. 3A). In our model, we simplified this by incorporating auxin-dependent expression of YUCCA4 specifically in the pericycle:
(9)

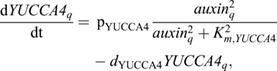
where *p*_YUCCA4_ is the production rate, *K*_m,YUCCA4_ the half-saturation constant of auxin dependent transcription, and *d*_YUCCA4_ the degradation rate.

Third, it has been suggested, that not only auxin levels, but also auxin signalling contributes to the early stages of LR formation (Moreno-Risueno et al., 2010). We thus assume that in addition to the default-expressed ARFs (not modelled explicitly), auxin elevation may cause additional ARF expression in elongation and DZ pericycle cells, which may subsequently contribute to auxin-dependent gene expression (Fig. 3A).

Following our previous work (Mähönen et al., 2014), for ARF-TIR1-IAA-auxin dynamics we write:
(10)

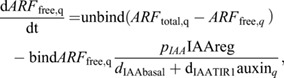
which can be derived from
(11)

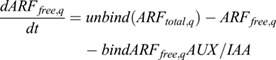
and
(12)

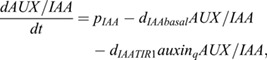
by taking a quasi steady state assumption for *AUX*/*IAA*.

Note that although the thus applied modelling of (additional) auxin signalling dynamics is similar to approaches applied by others, it is simplified in the sense that, compared with, for example, the work of Middleton et al. (2010), we do not model ARF-dependent AUX/IAA expression resulting in negative feedback nor ARF and IAA hetero- and homo-dimerization (e.g. Farcot et al., 2015). Clearly, with more complex models of auxin signalling more complex phenomena, such as oscillations within the auxin signalling network itself may occur (Middleton et al., 2010), but for conditions where this is not the case these more complex models can be directly mapped to the simpler approach we followed here. In light of the already highly complex, parameter-rich model developed here, we therefore decided to apply a more simple modelling approach for the auxin signalling.

For ARF expression we write:
(13)

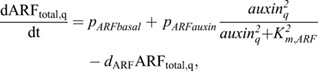
where *unbind* is the dissociation rate of the ARF-AUX/IAA complex, *bind* the association rate between ARF and AUX/IAA, *pIAA* the AUX/IAA production rate, *d*_*IAAbasal*_ the baseline AUX/IAA degradation rate, *dIAATIR*1 the auxin-dependent TIR1/AFB-induced extra degradation of AUX/IAA, *p*_*ARFbasal*_ the baseline ARF production rate, *p*_*ARFauxin*_ the auxin-dependent ARF production rate *K*_*m*,*ARF*_ the auxin-level at which auxin-dependent ARF production is half-maximal, and *d*_*ARF*_ the ARF degradation rate. *IAAreg*, which is here set as a constant at 1, enables the incorporation of additional regulatory effects impacting IAA levels, which we will use in a later section when incorporating lateral inhibition signalling.

To ensure that a secondary rise in auxin signalling occurs, auxin-dependent increases in LAX3, YUCCA4 and ARF expression are assumed only to occur beyond a cellular differentiation level of 80 (i.e. the differentiation factor described in Eqn 6 should be >80) (Fig. 3A).

#### Time-integrated positive-feedback model

As a next model extension, we incorporate a variable *EpiO* representing an auxin-dependent chromatin open state, which, through its slow dynamics, enables time-integrated tracking of the auxin signalling experienced by cells:
(14)

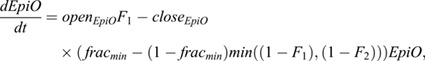

(15)

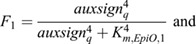

(16)

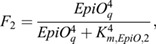
where *open*_*EpiO*_ is the maximum rate of chromatin opening, *close*_*EpiO*_ is the maximum rate of chromatin closing, and *frac*_*min*_ is the minimum fraction of the chromatin closing rate that is always effective. Chromatin opening rate is a saturating function *F*_1_ of auxin signalling, reaching its half maximum rate at an auxin signalling level of *K*_*M*,*EpiO*,1_. Chromatin closing is inhibited by both auxin signalling [(1−*F*_1_)] and chromatin open state [(1−*F*_2_)], assuming that beyond a certain threshold level chromatin open state leads to transcriptional activity and transcription limits chromatin closing.

This chromatin open state gates in a non-linear manner the auxin signalling-dependent transcription of LAX3, YUCCA4 and ARF:
(17)

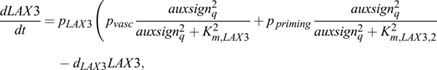

(18)

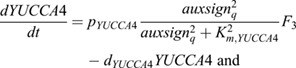

(19)

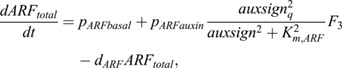
with
(20)

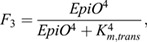
where *K*_*m*,*trans*_ is the EpiO level at which half-maximum auxin-dependent transcription is possible.

The non-linear function *F*_3_ implements the assumption that for effective transcription a sufficiently open chromatin structure is required. As before, the auxin signalling-dependent induction of LAX3, YUCCA4 and ARF is repressed below a differentiation level of 80.

Note that although for YUCCA4 and ARF the gene expression equations are the same as before except for the EpiO-dependent gating of their auxin induction, for LAX3 we used a slightly adjusted equation compared with before. Here, we split up the auxin-dependent gene expression into a part, *p*_*vasc*_, representing general vascular expression that is not related to LR priming and hence independent of EpiO gating and a part, *p*_*priming*_, that is related to priming and LR formation and hence is EpiO gated.

#### Lateral inhibition

To incorporate the ARF-LBD-TOLS2-RLK7-PUCHI lateral inhibition pathway (Fig. 6A) in our model in a simplified manner (Fig. 6B), we make production of the TOLS2 signalling peptide directly dependent on free ARF levels and model its transport as simple cell-to-cell diffusion:
(21)

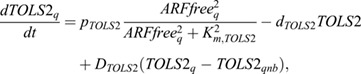
where *p*_*TOLS*2_ is the TOLS2 production rate, *K*_*m*,*TOLS*2_ the TOLS2 half-saturation constant, *d*_*TOLS*2_ the TOLS2 degradation rate and *D*_*TOLS*2_ the TOLS2 diffusion rate.

To model the repressive effect of TOLS2 signalling on auxin signalling by elevating IAA levels, as well as the protective effect of the cells’ own ARF levels against this inhibition of auxin signalling to create lateral inhibition, we replace the previously constant *IAAreg*=1 in the free ARF equation with:
(22)

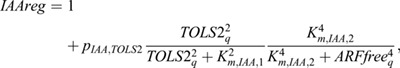
where *p*_*IAA*,*TOLS*2_ is the additional TOLS2-mediated IAA production *K*_*m*,*IAA*,1_ the half-saturation constant for TOLS2-mediated IAA upregulation and *K*_*M*,*IAA*,2_ the half-saturation constant for free ARF-mediated repression of IAA upregulation.

### Cellular dynamics

#### Zonation

As stated above, we superimpose locations and boundaries of the different developmental zones. Cells for which the lower cell boundary is at a distance of 514 μm or less from the root tip are part of the meristem, and cells at a distance between 364 and 514 μm from the root tip are part of the transition zone, the distalmost region of the meristem in which cells still grow cytoplasmatically but no longer undergo divisions.

For the faster maturing LR cap, the MZ ends at 314 μm from the root tip. After this, cells enter the EZ, and when their differentiation level exceeds a threshold transition to the terminal DZ occurs.

#### Cell growth, division and expansion

In both the meristem and transition zone, cells undergo slow cytoplasmic growth. As for our previous models, cell growth, division and expansion are only implemented in the rectangular cells positioned above the ending of the curved part of the LR cap and columella. Growth of the QC, columella and lower LR cap cells is thus ignored in our model. Because cells are modelled on a discrete grid, cells can only show growth by periodic, discrete addition of a row of grid points.

Previously, we simply calculated the time until the next addition of a grid row to a cell as the inverse of the cellular growth rate, with the latter being the product of the per micrometre growth rate (see below) and the discrete, grid-based height of the cell. As a consequence, each time a grid row was added and the discrete height of the cell increased by one a discrete jump in overall cellular growth rate also occurred, whereas within this time interval growth rate stayed equal. Although this effect is minor when cells in different cell files have the same height and positioning, this becomes problematic if, for example, one big cell is neighboured by two cells half its height. Under these conditions, because the larger cell will overall grow more rapidly it will add grid rows and achieve an acceleration of growth rate at earlier time points. This will cause it to become out of sync and start sliding relative to its neighbours, whereas on the per micrometre basis the one big and two small cells should grow equally fast. To minimize this unrealistic sliding and ensure a continuous rather than abruptly changing cellular growth rate, we now endow cells also with a continuous valued cell length that is continuously updated based on the per micrometre growth rate. When this continuous valued cell length exceeds the integer, grid-based cell length by a value of 1 a new row of grid points is added to the cell and the integer cell length counter is updated.

Upon doubling their original cell size, cells in the MZ divide, with daughter cells inheriting the PIN and AUX/LAX prepattern levels and polarity patterns of their mother cells. As cells at later stages enter the EZ and DZ, cellular PIN and AUX/LAX prepatterns are adjusted to the zone-specific patterns.

In the EZ, cells undergo rapid vacuolar expansion. Again, this is implemented by the periodic addition of a row of grid points to increase cell length. As growth now arises from an increase in vacuolar volume rather than an increase in cytoplasmic volume no dilution of protein levels is assumed to occur.

The per micrometre growth rates are computed from the duration of the division cell cycle or expansion duration (Table 1). For example, as cells need to double in size prior to division, solving 2*L*=*Le*^*rt*^ for the given division duration *t*=*cellcycle* gives us the growth rate *r* for cell division-related growth.

#### Cell differentiation

Upon entering the EZ, cells also start their initial differentiation. We model this through a generalized differentiation factor Diff undergoing a constant rate of differentiation *p*_*Diff*_ as well as differentiation decay *d*_*Diff*_. The latter is incorporated to enable dedifferentiation of partly differentiated cells upon a perturbation that changes the identity of the developmental zone in which the cell is located (i.e. reversal from EZ to meristem as a result of, for example, elevation of PLETHORA levels):
(23)

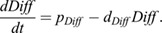
As cells exceed a threshold Th_Diff_>85 they cease elongation and enter the zone of terminal differentiation where further length increase ceases.

#### Semi-homogeneous cellular division rates

Recently, we demonstrated that LR priming arises from the synergy between root tip cell division dynamics and auxin transport (van den Berg et al., 2021). In this previous study, to be able to compare model predictions closely with data, we strived for maximally realistic root tip cell division dynamics, incorporating a gradient of division rates from the QC via stem cells to transit-amplifying cells as well as differences in cell size, division rates and zonation between vasculature and other cells. These detailed division dynamics resulted in realistic, semi-regular priming dynamics. Here, to study the translation of priming into PBS formation, we need to be able to discern transients in model dynamics from variations in priming dynamics between individual priming events in order to establish when stable PBS formation occurs. Therefore, we minimized variation in priming dynamics in our simulations by ignoring differences in cell division rates between the QC, stem cells and transit-amplifying cells or cell types within the meristem. To avoid artificial synchronized division of the entire meristem, deterministic position-based small variations in division rates were applied. This was carried out as follows: if (mean_j%3==0), divisionrate=average division rate; else if (mean_j%3==1), division rate =average division rate +5%; else if (mean_j%3==2), division rate=average division rate −5%, where mean_j is the longitudinal coordinate of the centre of the cell after its origination from the division of the mother cell (i.e. it is determined once for each cell, not constantly updated).

Only in a subset of simulations (Fig. 2E,F, Fig. S2) did we incorporate cell height differences in the meristem (vasculature 12 μm, epidermis and cortex 10 μm, pericycle and endodermis 8 μm), as well as differences in the onset of the transition zone between different cell types [as done previously by van den Berg et al. (2021)] to obtain more realistic staggered cell patterns.

#### Constant-sized finite simulation domain

In our model, we make use of a constant, finite-sized simulation domain. For the simulations used in this study, with a spatial integration step of Δ*x*=2 μm, the simulation domain measures 141 times 1516 grid points or 282 times 3032 μm.

Within this domain, we keep the root tip at a fixed location in the bottom of the simulation domain. Hence, as a result of formation, division and growth of younger cells below them older cells are displaced towards the top of the simulation domain. If the wall above the apical membrane of a cell reaches the topmost position of the simulation domain, this cell is removed from the simulation.

Additionally, to mimic the shedding of LR cap cells, the topmost LR cap cells are removed when the basal membrane of that cell exceeds the position where other non-LR cap cells leave the meristem and enter the EZ (at 314 μm from the root tip).

### Approximating 3D tissue architecture

In our simulations we model a 2D longitudinal cross-section of the *Arabidopsis* root tip. Given that the *Arabidopsis* root tip, and particularly the vasculature, is not perfectly radially symmetric, the orientation of this cross-section was chosen such that it encompasses the xylem poles and neighbouring pericycle, which are for priming essential tissues.

However, by taking such a 2D approach, the radial auxin fluxes that occur in the 3D plant tissue are ignored, whereas in a previous modelling study that used radial instead of longitudinal cross-sections these have been previously shown to play an important role in the symmetry breaking between LR prebranch/initiation sites (el-Showk et al., 2015). Therefore, to at least partially emulate the 3D nature of true plant roots we incorporated radial auxin flows between laterally symmetric positions in the root.

In the default model settings, we only incorporated a passive auxin exchange between laterally symmetric wall grid points. To achieve this, we added the following term to the auxin differential equations for the wall grid points:
(24)

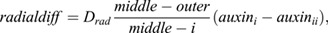
where *i* is the coordinate of the left and *ii* the coordinate of the laterally symmetric right position, *D*_*rad*_ the radial diffusion rate, *middle* the coordinate of the root tip middle and *outer* the coordinate of the leftmost wall of the root tip. By incorporating 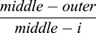, we inversely scale the rate of passive diffusion between laterally symmetric positions with their radial distance. All simulations except the one shown in Fig. S8A incorporate these radial flows.

In the simulation in Fig. S8B, in addition to the above-described passive auxin flow between laterally symmetric wall grid points, we added the following radial exchange between non-wall grid points:
(25)

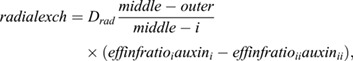

(26)

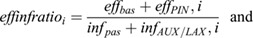

(27)

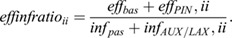


Here, the *effinfratio* terms reflect that the fraction of cell internal auxin available for exchange depends on the ratio between cell efflux and influx: the higher that ratio, the more easily the auxin is lost to the outside where it can be exchanged with other cells. As for the passive radial fluxes, the transport rate is scaled with the inverse of the distance between the symmetrical positions.

### Staggered cell wall settings

To investigate whether the priming dynamics we observe in our root tip model rely on the somewhat artificial parallel cell wall positioning between neighbouring cell files in our model, we performed alternative simulations. Here, we incorporated the fact that for the vasculature the EZ starts closest to the root tip, whereas for the pericycle it starts furthest from the root tip, as well as differences in cell height (epidermal and endodermal cells 10 μm, vasculature cells 12 μm, and other cells 8 μm). Combined, this results in staggered cell wall positioning between cell files.

Although we adjusted cell growth dynamics to minimize non-realistic sliding of cells past one another (see ‘Cell growth, division and expansion’ section of Materials and Methods), sliding between neighbouring cells of distinct heights cannot be fully prevented in our model, in which cells consist of discrete number of grid cells. As sliding effects accumulate with distance from the root tip, results from the staggered cell wall model are still acceptable close to the root tip, yet deviate more further from the root tip. Therefore, we used these alternative simulations solely to confirm that priming dynamics, occurring relatively close to the root tip, do not rely on cell wall positioning, yet refrain from using these simulations to study the PBS formation occurring further away from the root tip as we cannot exclude sliding effects impacting auxin exchange between neighbouring cells there.

Proper simulation of root growth dynamics, particularly in more realistic root topologies with staggered differentially sized cells between cell files, awaits the development of modelling formalisms incorporating the mechanics of symplastic root growth. Although various efforts in this direction exist (Fozard et al., 2013, 2016; Marconi et al., 2021; Weise and Ten Tusscher, 2019), to our knowledge none has yet managed to combine a realistic modelling of the particularly challenging, highly anisotropic elongation dynamics of root cells in models simultaneously describing cell-based gene expression and cell- or finer-resolution-based auxin dynamics.

### Model parametrization

#### Baseline auxin dynamics

Values of parameters involved in baseline auxin production (*p*_*auxin*,*baseline*_) and degradation (*d*_*auxin*_), additional cell type-dependent auxin production (*celltypefactor*), gene expression-mediated upregulation of auxin production (*yucca*4*factor*) and auxin transport rates and directions (intracellular and intra-apoplastic diffusion *D*_*cell*_, *D*_*wall*_, PIN-mediated export *p*_*PIN*_, ABC-mediated export *eff*_*basal*_, AUX1-mediated import *p*_*AUX*/*LAX*_, baseline import *inf*_*pas*_) are taken as equal or close to values from previous well-established model studies, dating back as far as the seminal study by Grieneisen et al. (2007). For these parameter settings, models were shown to produce qualitatively realistic and robust auxin patterns (van den Berg et al., 2016, 2021; Grieneisen et al., 2007; Mähönen et al., 2014; Rutten and Ten Tusscher, 2021; Salvi et al., 2020) consistent with shoot cut experiments (Grieneisen et al., 2007), capable of simulating tropic responses (van den Berg et al., 2016; Mähönen et al., 2014) consistent with more recent insights on the importance of root localized auxin production (van den Berg et al., 2021; Rutten and Ten Tusscher, 2021; Salvi et al., 2020), and consistent with experimentally observed changes in auxin and meristem dynamics in response to mutations (van den Berg et al., 2021; Salvi et al., 2020) . Under these parameter settings, the basic priming dynamics shown in Fig. 2 are obtained.

#### Gene expression, differentiation and epigenetic state

Importantly, although root tip auxin patterning is extensively measured, these measurements are of a relative rather than absolute nature, showing, for example, how much auxin levels in the QC are higher than in the more shootward boundary of the meristem, or how much auxin levels change in response to a mutation or treatment, rather than providing actual concentrations. Therefore, the above models as well as our current baseline model qualitatively reproduce root tip auxin patterning and dynamic changes therein, yet absolute simulated concentration levels are arbitrary, i.e. a similar qualitative match between experiments and model could be achieved if concentrations at all locations were increased tenfold or decreased fivefold. As a consequence, activation thresholds (Km) for the auxin signalling-dependent activation of AUX1, LAX3, YUCCA4, ARF, TOLS2 and EpiO are parametrized relative to the modelled auxin gradient such that activation occurs in the right spatial domains and under the right conditions. Thus, for example, the Km for AUX1 is higher than that for basal LAX3, resulting in AUX1 expression mostly in the meristem where auxin levels are high and LAX3 expression instead being able to extend more shootward. Similarly, the Kms for additional priming related LAX3 activation, YUCCA4, TOLS2 and ARF/EpiO are chosen relatively high, such that activation only occurs in response to priming. This ensures the model reproduces the observed root tip-wide general AUX1 and LAX3 patterns, as well as the observed localized induction of AUX1, LAX3, YUCCA4 and TOLS2 under PBS formation, and the here hypothesized localized induction of ARF and EpiO under PBS formation. It is noteworthy that in the EpiO time-integrated model, owing to the more targeted induction, higher saturation constants for LAX3 and YUCCA4 suffice to obtain significant upregulation of gene expression under PBS formation.

Additionally, in the absence of data on absolute levels of gene expression, we apply for all genes a maximum expression level of 100, by choosing a 1:100 ratio between degradation and maximum production rates (AUX1, LAX3, YUCCA4, TOLS2, Diff) or using a constant level of 100 (PIN), as done previously (Mähönen et al., 2014). For LAX3, which has in addition to its priming-related upregulation of expression a broad approximately constant expression in the vasculature of the EZ and DZ, in addition to the maximum priming-driven expression rate a constant baseline/vascular expression rate was also incorporated. Similarly, maximum opening and maximum closing rates for EpiO were taken to generate an EpiO level of 100, with reduced opening or reduced closing resulting in lower or higher levels, respectively. Next, decay rates between 0.0001 and 0.00015 s^−1^ were used, generating half times of 1.3-1.9 h to enable significant (de)activation of the involved genes (AUX1, LAX3, YUCCA4, ARF, TOLS2) and epigenetic state (EpiO) on the several-hour timescale relevant for priming dynamics.

As an exception to the maximum level of 100, for ARF a maximum level of 300 was chosen in order to obtain an increase in auxin signalling capacity that is sufficient to lead to stable PBS formation. Additionally, we apply a baseline expression of 10 for ARF, necessary for initiating the positive feedback of more auxin, by freeing of ARF from AUX/IAA, leading to more ARF expression and hence more auxin signalling capacity. Similarly, for TOLS2 a maximum production rate of 1000 was chosen to compensate for its production in a limited number of cells and subsequent transport to a larger number of cells. Finally, for the TOLS2 peptide the diffusion constant was chosen such that, given the applied production and degradation rates, effective inhibition of neighbouring sites would ensue in a timely manner.

#### Growth dynamics

For cellular expansion, a timescale of 7 h was used, in agreement with our previous experimental observations (Mähönen et al., 2014). For cellular division, a gradient of cell cycle durations from 60 h in the QC to 20 h in the shootward end of the MZ was recently observed (Rahni and Birnbaum, 2019). Additionally, divisions were reported to be significantly more rapid in the stele with a cell cycle duration of around 16 h compared with outer tissues (24 h). Given the importance of vascular divisions for our priming dynamics, we therefore incorporated in our previous reflux-and-growth priming model cell cycles from 41 h tapering off to 12 h over the first four cells of the QC and more rapid transit-amplifying divisions with cell cycles from 9-11 h depending on the simulation. In the current model, to generate regular, more easily comparable priming dynamics, all cells divide synchronously, except for a limited 10% extent of noise on their cell cycle duration (see ‘Semi-homogeneous cellular division rates’ section in Materials and Methods). Under these more homogeneous conditions, a reduction in priming frequency occurs. To compensate for this, we used the shorter cell cycle duration of 9 h, ensuring resulting priming frequencies at least fit within the upper range of experimental observations (De Smet et al., 2007).

### Kymographs

Kymographs were generated as described previously (see figure 1B and movie 1 in van den Berg et al., 2021). Briefly, we draw a single grid point-wide vertical line through the middle of the left pericycle cell file, plotting auxin levels along the length of this 1D line using a colour gradient to represent auxin levels. By plotting auxin levels along this 1D line every 100 s and concatenating these 1D lines in the vertical direction, a space-time plot of auxin dynamics is generated.

### Numerical integration and run-time performance

Similar to previous root tip modelling studies by us and others (van den Berg et al., 2021; Grieneisen et al., 2007; Mähönen et al., 2014), we used an alternating direction semi-implicit integration scheme for the grid-level auxin partial differential equations (Peaceman and Rachford, 1955), using integration steps of 0.4 s and a spatial integration step of Δx=2 μm. For the cell-level gene expression, ordinary differential equation standard Euler forward integration was applied. The code of the model was written in C++, simulations were run on 24- to 36-core workstations with Intel Xeon E5-2687W processors, resulting in a typical run-time of ∼12 h for a simulation representing 6 days of plant growth.

## Supplementary Material

10.1242/develop.200927_sup1 Supplementary information
