# Supplementary material for: Complementary roles for auxin and auxin signalling revealed by reverse engineering lateral root stable prebranch site formation
Source: Development. 2022 Nov 21;149(22):dev200927. doi: 10.1242/dev.200927 (PMC9793420; doi:10.1242/dev.200927)
Supplement: Supplementary information [file develop-149-200927-s1.pdf]

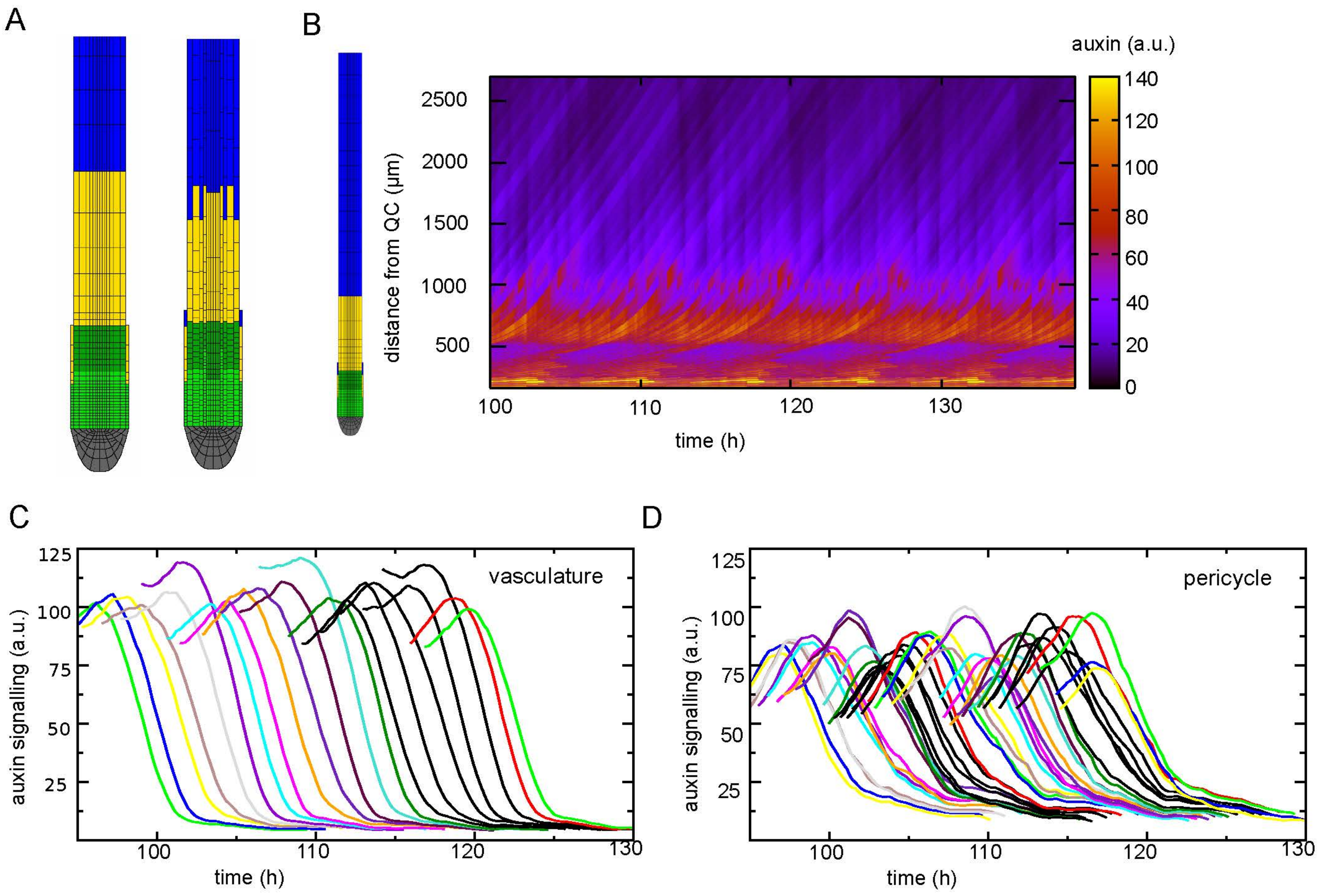

Fig. S1. Priming under staggered cell wall positioning. A) comparison of cell layout in case of similar cell sizes and similar developmental zone boundaries resulting in parallel cell wall locations across cell files (left) and in case of different cell sizes and transition zone boundaries resulting in staggered cell wall locations across cell files (right). B) Kymograph of pericycle auxin dynamics for the staggered cell wall model. All other settings are equal to those applied in Fig 2. C,D) Vascular (C) and pericycle (D) auxin dynamics as a function of time. E,F) Vascular (E) and pericycle (F) auxin dynamics as a function of distance from the root tip. Note that due to cell size differences, within the same time window a different number of cells is traced for these two tissue types, and that larger vascular cells being next to a variable number of variably positioned pericycle cells result in more irregular priming dynamics.

Development • Supplementary information

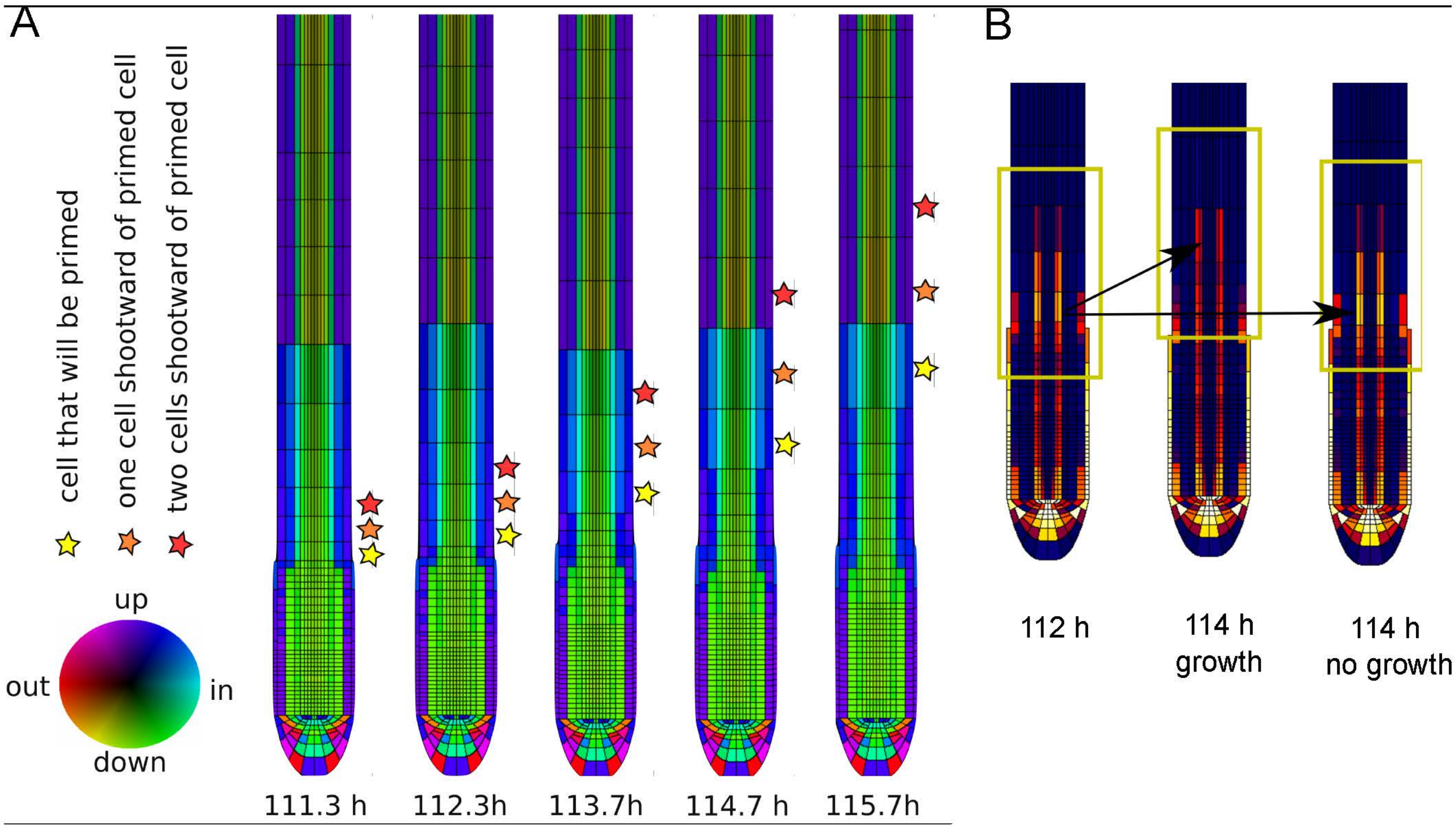

Fig. S2. Auxin flux and growth displacement. Temporal sequence of auxin flux direction snapshots from the early (111.3 h) to late stages (115.7 h) of a single priming event. D) Auxin signalling during early (112 h), and later stages of priming (114 h) in presense or absence of continued growth.

Development • Supplementary information

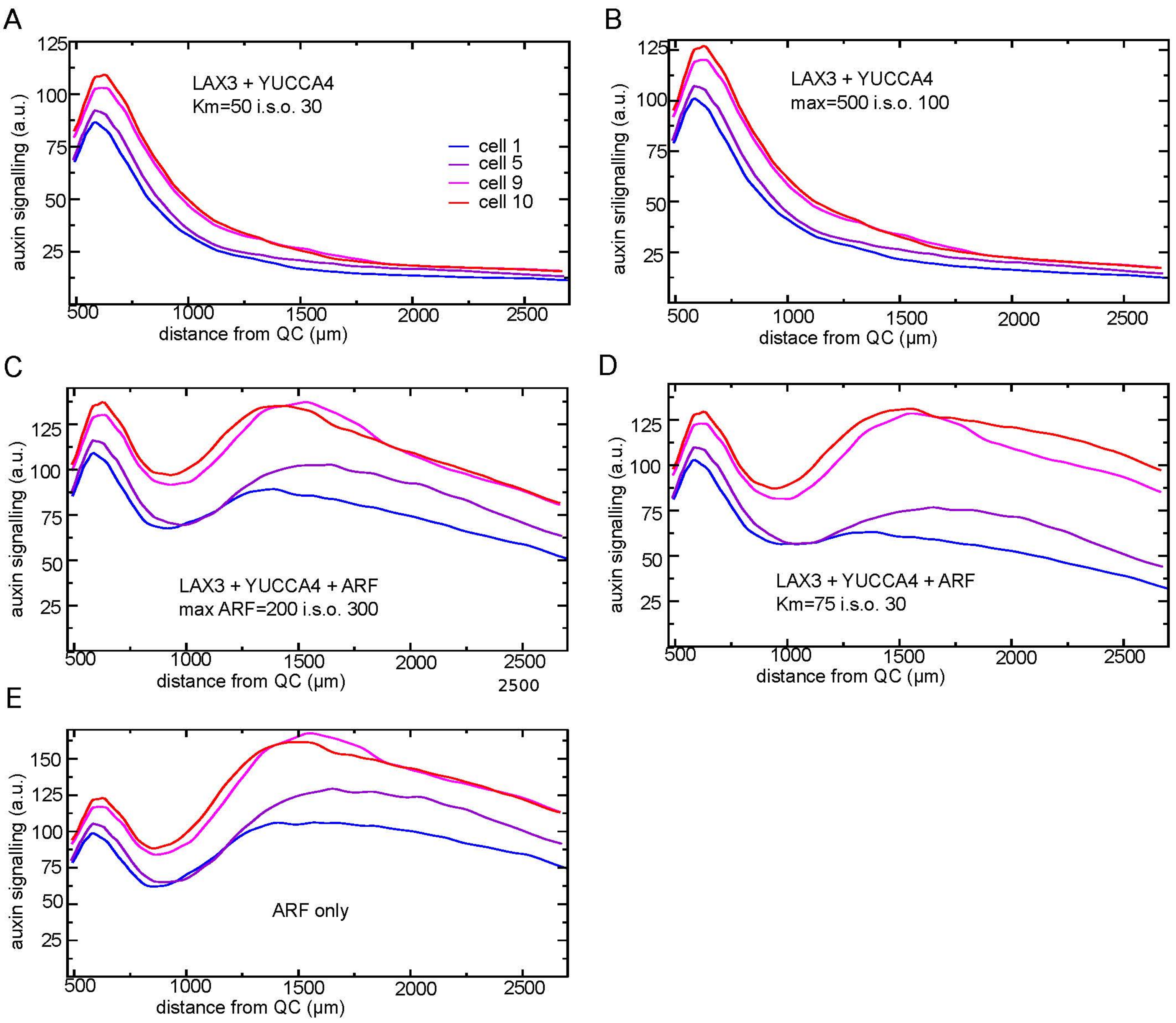

Fig. S3. Positive feedback for alternative parameter settings. A) Pericycle auxin signalling for only LAX3 +YUCCCA4 positive feedback for Km=50 instead of Km=30 (Fig 3B), indicating limited effect of Km on effectiveness positive feedback. B) Pericycle auxin signalling for only LAX3 + YUCCA4 feedback for maximum expression 500 instead of 100 (Fig 3B), indicating limited effect of maximum expression on effectiveness positive feedback. C) Pericycle auxin signalling for LAX3+YUCCA4+ARF feedback for max ARF expression 200 instead of 300 (Fig

3C), indicating a quantitative effect but otherwise similar behavior. D) Pericycle auxin signalling for LAX3 +YUCCA4+ARF feedback for Km=75 instead of 30 (Fig 3C), indicating this does not significantly enhance differentiation between cells receiving different strengths of priming signal
yet reduces strength of the secondary auxin signalling response. E) Pericycle auxin signalling in absence of auxin signalling induced YUCCA4 and additional LAX3 expression.

Development • Supplementary information

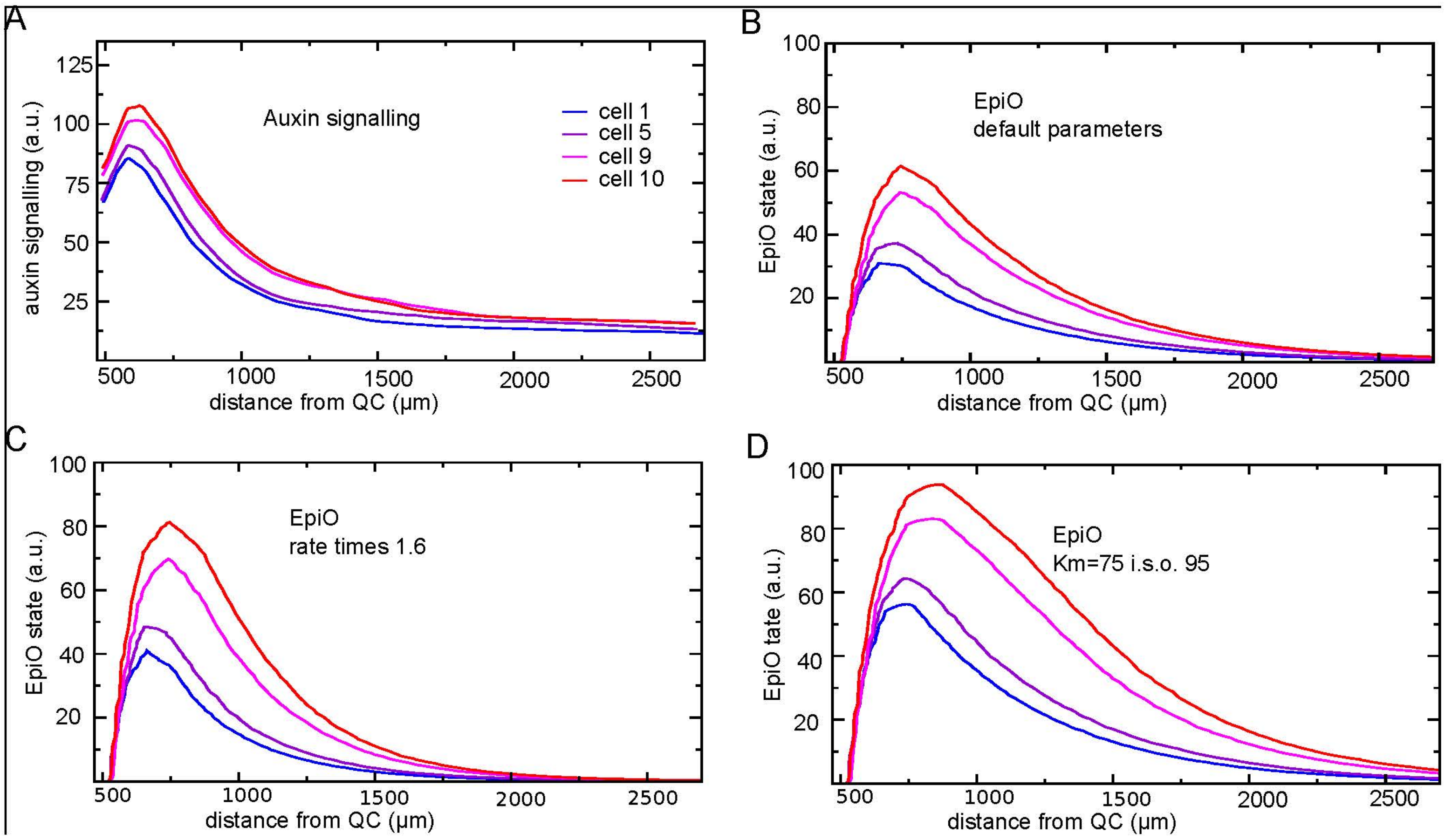

Fig. S4. EpiO dynamics for alternative parameter settings. A) Non-normalized auxin signalling dynamics as a function of distance corresponding to the normalized auxin dynamics shown in Fig 4C. B) Non-normalized EpiO dynamics corresponding to the normalized EpiO dynamics shown in Fig 4C. C) EpiO dynamics for non-default model settings where the production and degradation rates of EpiO where increased 1.6 fold. D) EpiO dynamics for non-default model settings where the Km for AuxinSignalling induced increase of EpiO state was changed from 95 to 75.

Development • Supplementary information

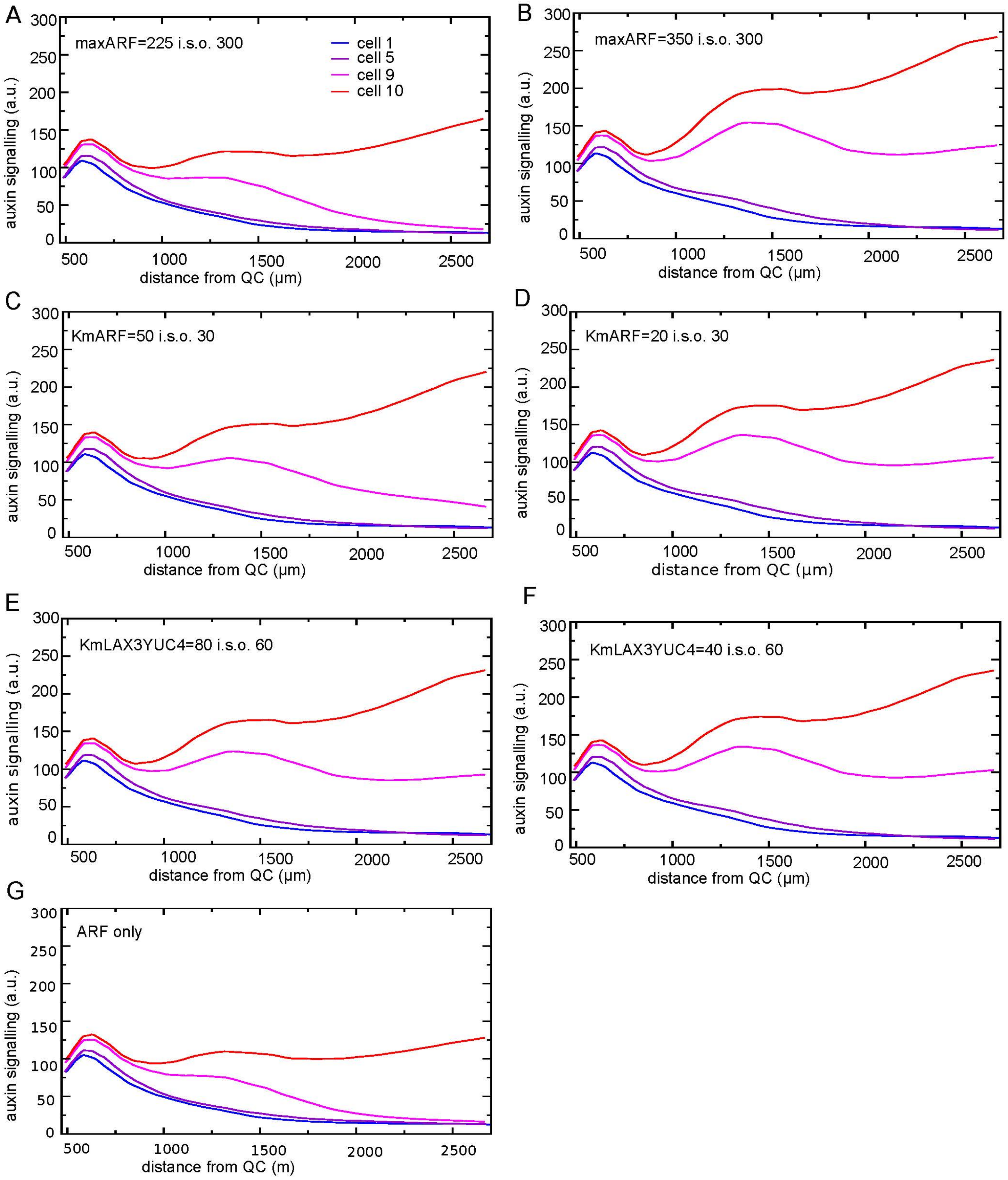

Fig. S5. Auxin signalling dynamics in EpiO + positive feedback model for alternative parameter settings. A, B) Decreased (A) and increased (G) maximum ARF expression, reduces respectively enhances overall auxin signalling levels. In case of a decrease formation of a second stable PBS does not occur. C,D) Increased (C) and decreased (D) Km for auxin signalling induced ARF expression reduces, respectively enhances overall auxin signalling levels. In case of an increase formation of a second stable PBS does not occur. E,F) Increased (E) and decreased (F) Km for auxin signalling induced LAX3 and YUCCA4 expression hardly effects overall auxin signalling dynamics. G) Auxin signalling dynamics in case only upregulation of ARF, but not LAX3 or YUCCA4 occurs.

Development • Supplementary information

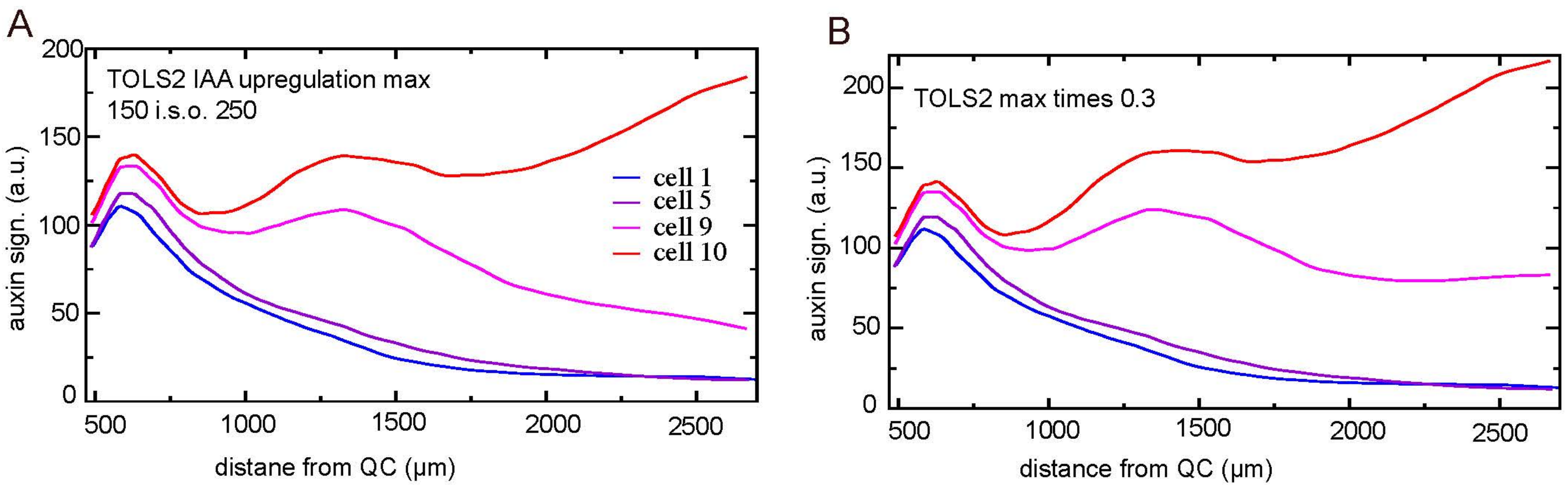

Fig. S6. TOLS2 signalling strength affects lateral inhibition efficiency. A) Reduction of the TOLS2 signalling effect on IAA induction with 40% reduces speed of secondary PBS inhibition. B) Reduction of the TOLS2 signalling level by 66.7% abolishes secondary PBS inhibition.

Development • Supplementary information

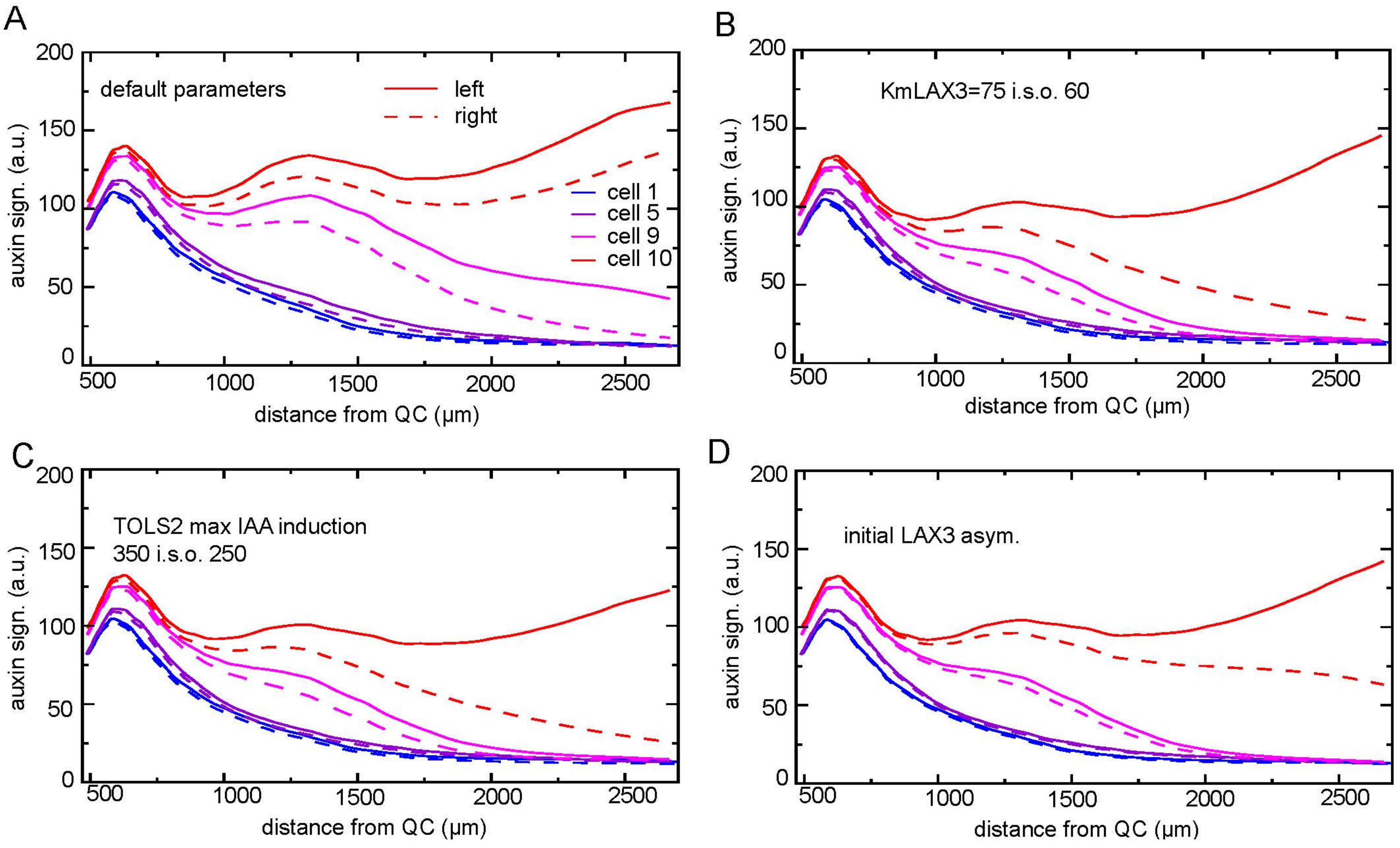

Fig. S7. Symmetry breaking for alternative parameter settings. A) Default model settings. B) Increase of LAX3 Km from 60 to 75. C) Increase of TOLS2 mediated maximum IAA induction by 40%. D) Initial assymmetry from 10% left-right difference in LAX3.

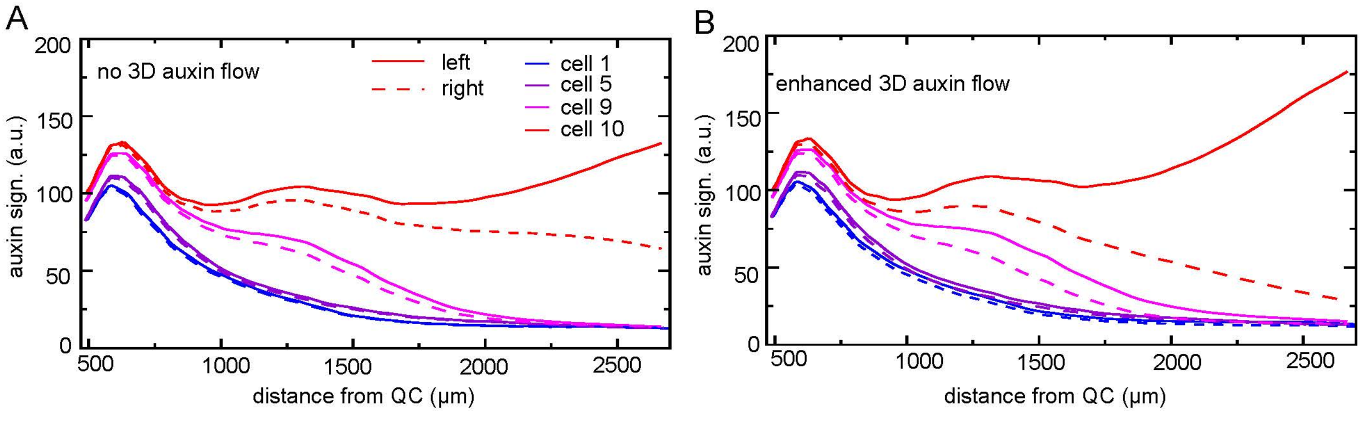

Fig. S8. 3D auxin flows enhance symmetry breaking. A) Absence of 3D auxin flows slows down repression of low AUX1 side PBS. B) Enhancement of 3D auxin flows enhances repression of low AUX1 side PBS. (For details see Methods)

Development • Supplementary information

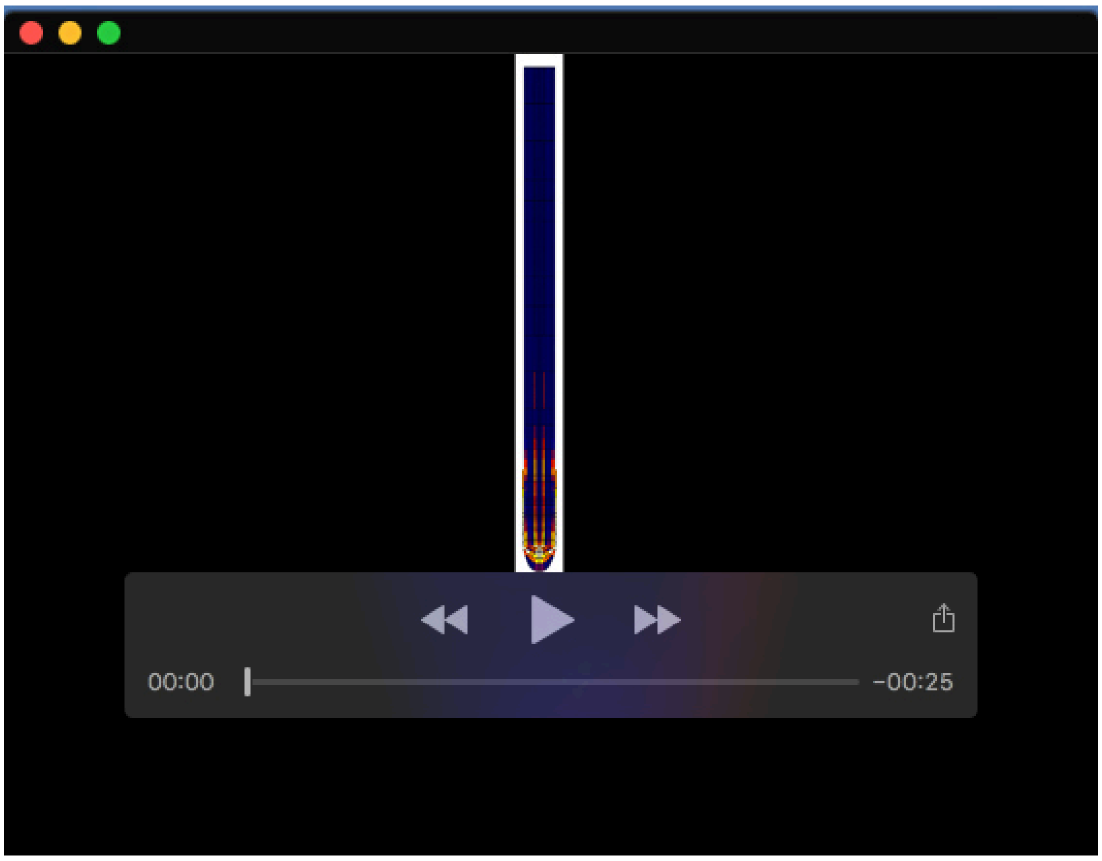

Movie 1. Dissipation of auxin signalling in a priming only simulation. The movie corresponds to the results shown in Figure 2.

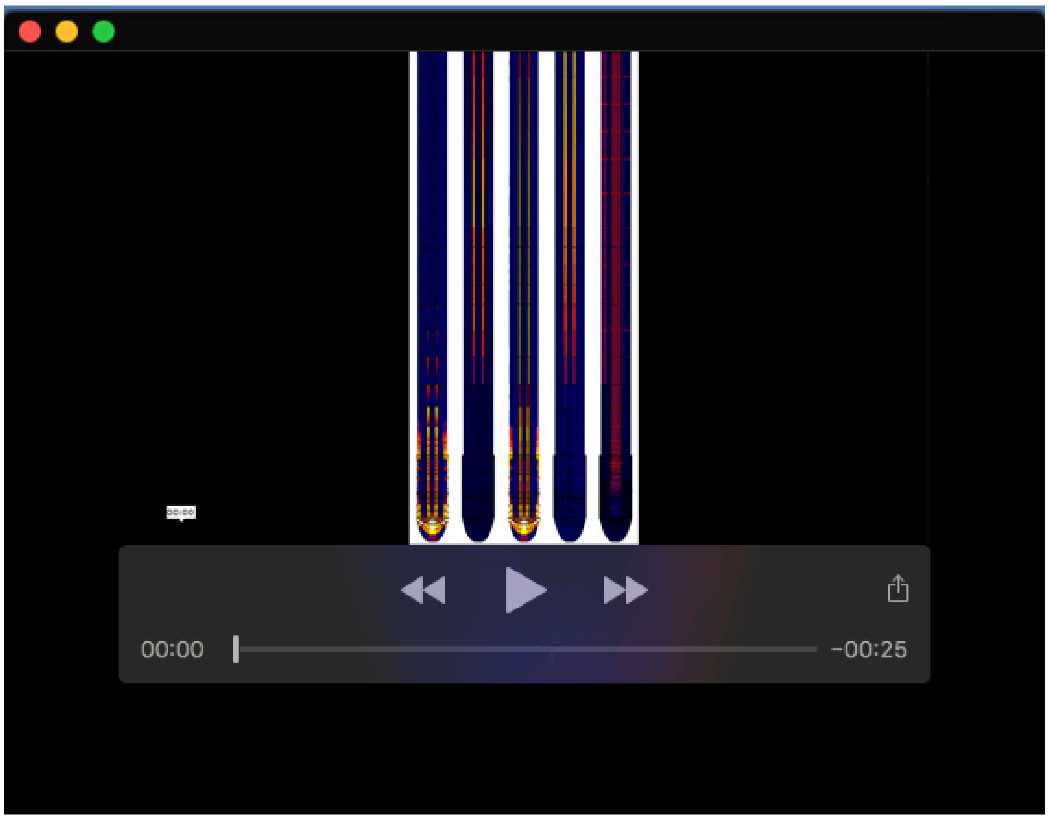

Movie 2. Auxin concentration, ARF, Auxin signalling, YUCCA 4 and LAX3 dynamics for the direct positive feedback model settings. The movie corresponds to the results shown in Figure 3C-D.

Development • Supplementary information

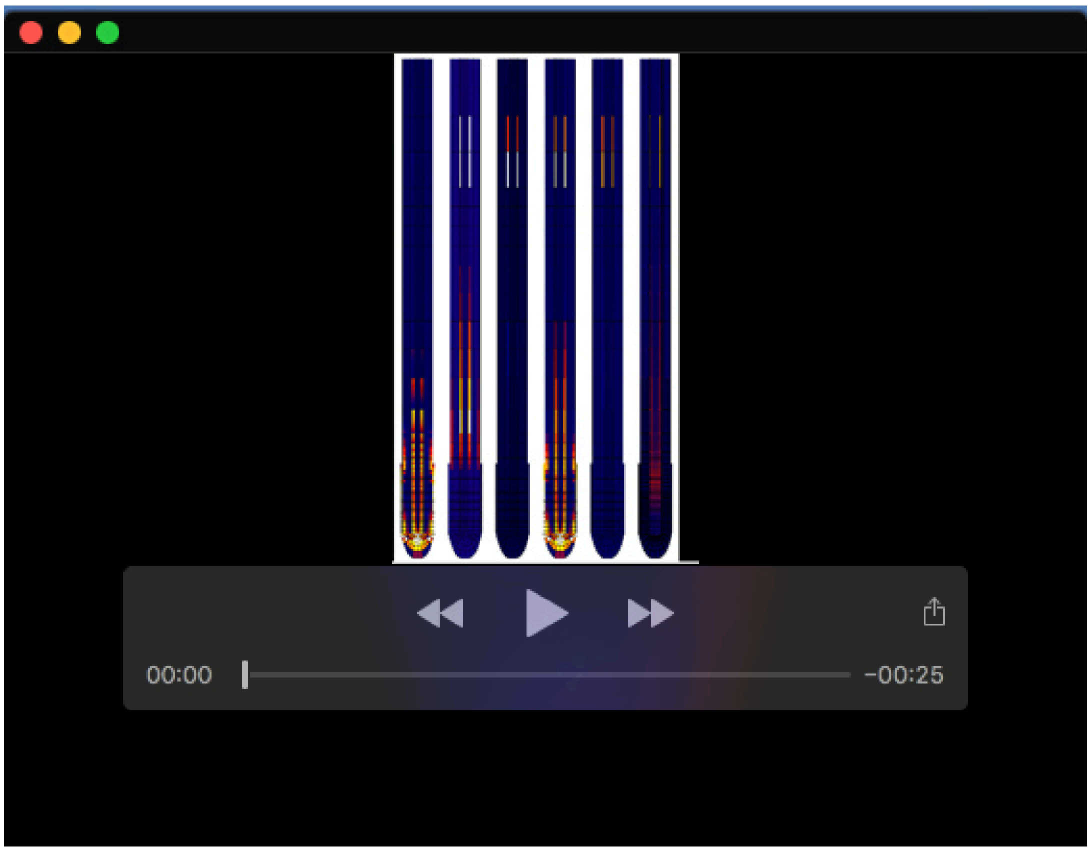

Movie 3 .Auxin concentration, EpiO, ARF, Auxin signalling, YUCCA 4 and LAX3 dynamics for the time integrated positive feedback model settings. The movie corresponds to the results shown in Figure 4 A-E and Figure 5 A-B.

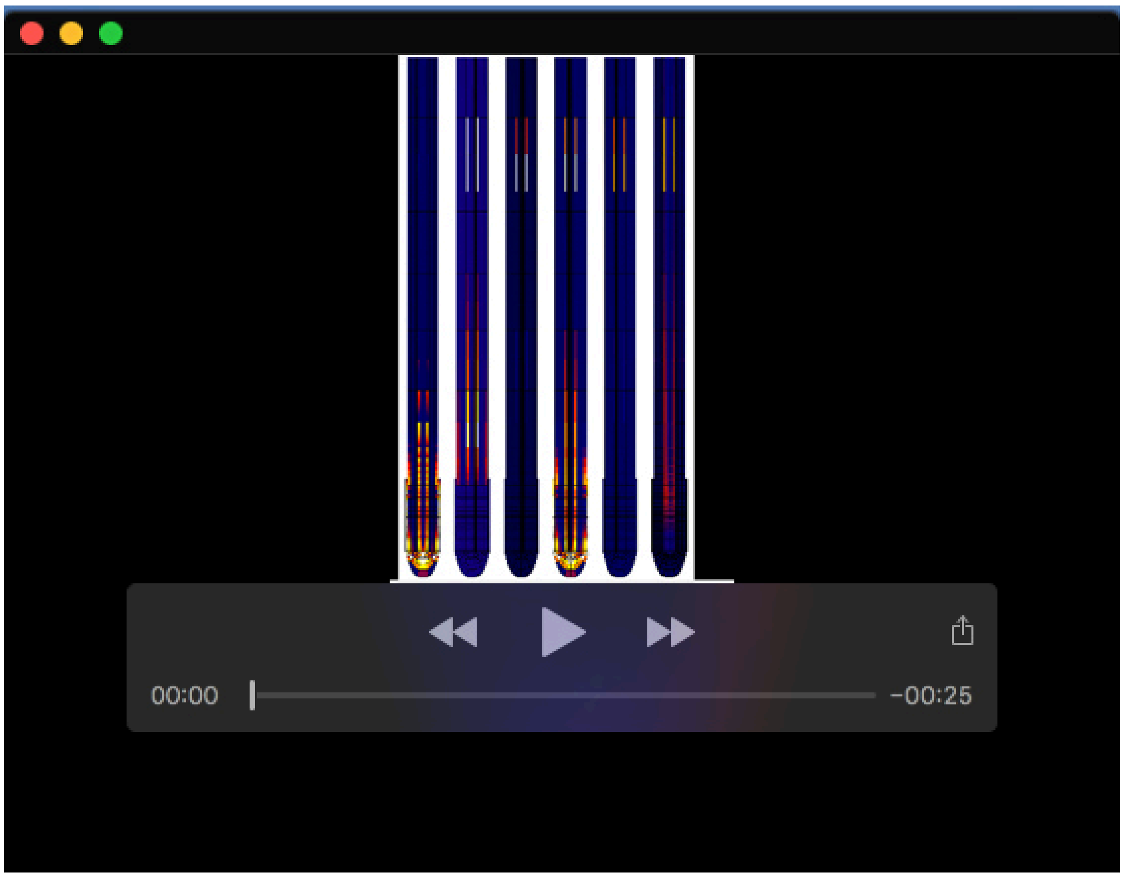

Movie 4. Auxin concentration, EpiO, ARF, Auxin signalling, YUCCA 4 and LAX3 dynamics for the time integrated positive feedback model settings incorporating TOLS2-RLK7-PUCHI mediated lateral inhibition. The movie corresponds to the results shown in Figure 6A-D.

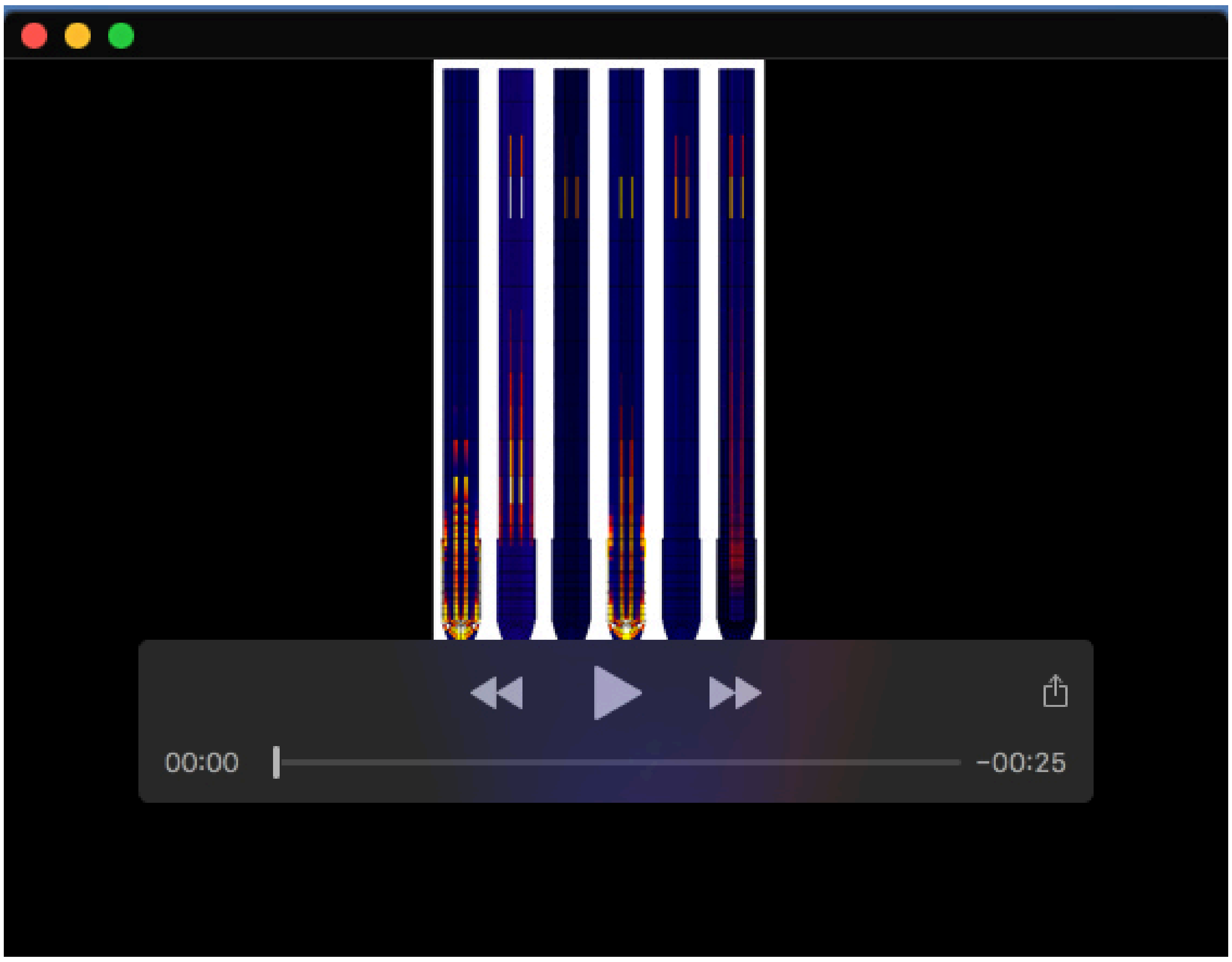

Movie 5. Auxin concentration, EpiO, ARF, Auxin signalling, YUCCA 4 and LAX3 dynamics for the time integrated positive feedback model settings incorporating TOLS2-RLK7-PUCHI mediated lateral inhibition and AUX1 induced symmetry breaking. The movie corresponds to the results shown in Figure 7A-B.
